# Intra-articular treatment of temporomandibular joint osteoarthritis by injecting actively-loaded meloxicam liposomes with dual-functions of anti-inflammation and lubrication

**DOI:** 10.1016/j.mtbio.2023.100573

**Published:** 2023-02-03

**Authors:** Yingqian Zhong, Yuyu Zhou, Ruoyi Ding, Luxiang Zou, Hongyu Zhang, Xiaohui Wei, Dongmei He

**Affiliations:** a Department of Oral Surgery, Ninth People’s Hospital, Shanghai Jiao Tong University School of Medicine, Shanghai, 200011, China; b Shanghai Key Laboratory of Stomatology & Shanghai Research Institute of Stomatology, Shanghai, 200011, China; c National Clinical Research Center of Stomatology, Shanghai, 200011, China; d School of Pharmacy, Shanghai Jiao Tong University, Shanghai, 200240, China; e State Key Laboratory of Tribology, Department of Mechanical Engineering, Tsinghua University, Beijing 100084, China

**Keywords:** Osteoarthritis, Liposomes, Lubrication, Intra-articular injection, Active drug loading

## Abstract

Temporomandibular joint (TMJ) osteoarthritis is a common osteochondral degenerative disease which can severely affect patient's mouth opening and mastication. Meloxicam (MLX), one of the most widely used non-steroidal anti-inflammatory drugs, is the main clinical therapy for the treatment of TMJ osteoarthritis. However, the clinical effect is greatly compromised because of its poor water solubility and high lipophilicity. In the present study, we developed an actively-loaded liposomal formulation, namely MLX-Ca(AC)_2_Lipo, using meglumine to enhance aqueous solubility and divalent metal (Ca^2+^) solution to improve encapsulation efficiency. By the formation of the nano-bowl shaped MLX-Ca precipitates inside the liposomes, MLX-Ca(AC)_2_Lipo successfully achieved an optimal encapsulation efficiency as high as 98.4% compared with previous passive loading method (60.6%). Additionally, MLX-Ca(AC)_2_Lipo maintained stable, and the slow drug release not only prolonged the duration of drug efficacy but also improved bioavailability. It was shown in the *in vitro* and *in vivo* tests that MLX-Ca(AC)_2_Lipo downregulated the synthesis of the inflammatory factors (such as prostaglandin-E2) and as a consequence reduced chondrocytes apoptosis and extracellular matrix degeneration. Furthermore, the intra-articular injection of MLX-Ca(AC)_2_Lipo enhanced bioinspired lubrication of TMJ, protecting the cartilage from progressive wear. In summary, MLX-Ca(AC)_2_Lipo with dual-functions of anti-inflammation and lubrication is a promising nanomedicine for the treatment of TMJ osteoarthritis by intra-articular injection.

## 1 Introduction

Temporomandibular joint (TMJ) osteoarthritis is a common chronic degenerative disease typically characterized by synovial inflammation, cartilage degeneration, and subchondral bone resorption or subchondral sclerosis [1]. The patients are accompanied with clinical symptoms such as limited mouth opening and severe chronic pain, which can impact mouth opening and masticatory functions [2]. It is known that an increased inflammation and mechanical pressure can deteriorate the development of osteoarthritis [[3], [4], [5]]. Generally, osteoarthritis is associated with the significantly increased friction and reduced lubrication of the joint, which causes irreversible and progressive destruction of the articular cartilage. The design of dual-functional biomaterials with controllable drug delivery and improved lubrication has been proved to be crucial in the treatment of osteoarthritis [[6], [7], [8]]. Therefore, it is very important to achieve local anti-inflammation and lubrication enhancement in order to prevent the progressive joint damage.

Meloxicam (MLX), one of the non-steroidal anti-inflammatory drugs (NSAIDs), is clinically used for the treatment of TMJ osteoarthritis based on the mechanism of selectively blocking cyclooxygenase-2 (COX-2) and meanwhile inhibiting synthesis of inflammatory factor prostaglandin-E2 (PGE2), which are considered to play a major role in the development of osteoarthritis [9,10]. Compared with other NSAIDs such as celecoxib, diclofenac, and etoricoxib, meloxicam has significant anti-inflammatory and analgesic effects with relatively low cost and toxicity. However, the absorption rate of meloxicam via oral administration is poor owing to its low aqueous solubility, and thus it is very difficult to achieve drug enrichment in the joint following oral delivery [11]. Consequently, various local meloxicam delivery systems have been developed to solve the problem [12], including carboxymethyl chitosan-methylcellulose-pluronic hydrogel [13], transdermal cationic liposomes [14], microemulsion [15], polymeric nanoparticles [16,17] and biodegradable gelatin microsphere [18]. To the best of our knowledge, to date there are no studies reported on the intra-articular application in TMJ because the aqueous environment of synovial fluid can lead to rapid drug release and decomposition.

It has been shown from previous studies that liposomes can prolong drug efficacy, improve drug bioavailability, achieve tissue/cell targeting, and also promote lubrication response [[19], [20], [21], [22]]. Recently, several liposome formulations of curcumin, rapamycin, and diclofenac sodium have been developed to enhance the inhibition of pro-inflammatory factors for the treatment of osteoarthritis [[23], [24], [25]]. However, these reported meloxicam liposomes are designated for transdermal application and mainly constructed by passive drug loading method [14,15], where the anti-inflammatory drug is loaded in liposome formation process and thus lack of sufficient encapsulation efficiency. Furthermore, no report has been available on the intra-articular injection of meloxicam liposomes owing to the relatively low drug loading capacity, poor storage stability, and potential risk of burst release by the passive loading method [26,27].

**Scheme 1:**
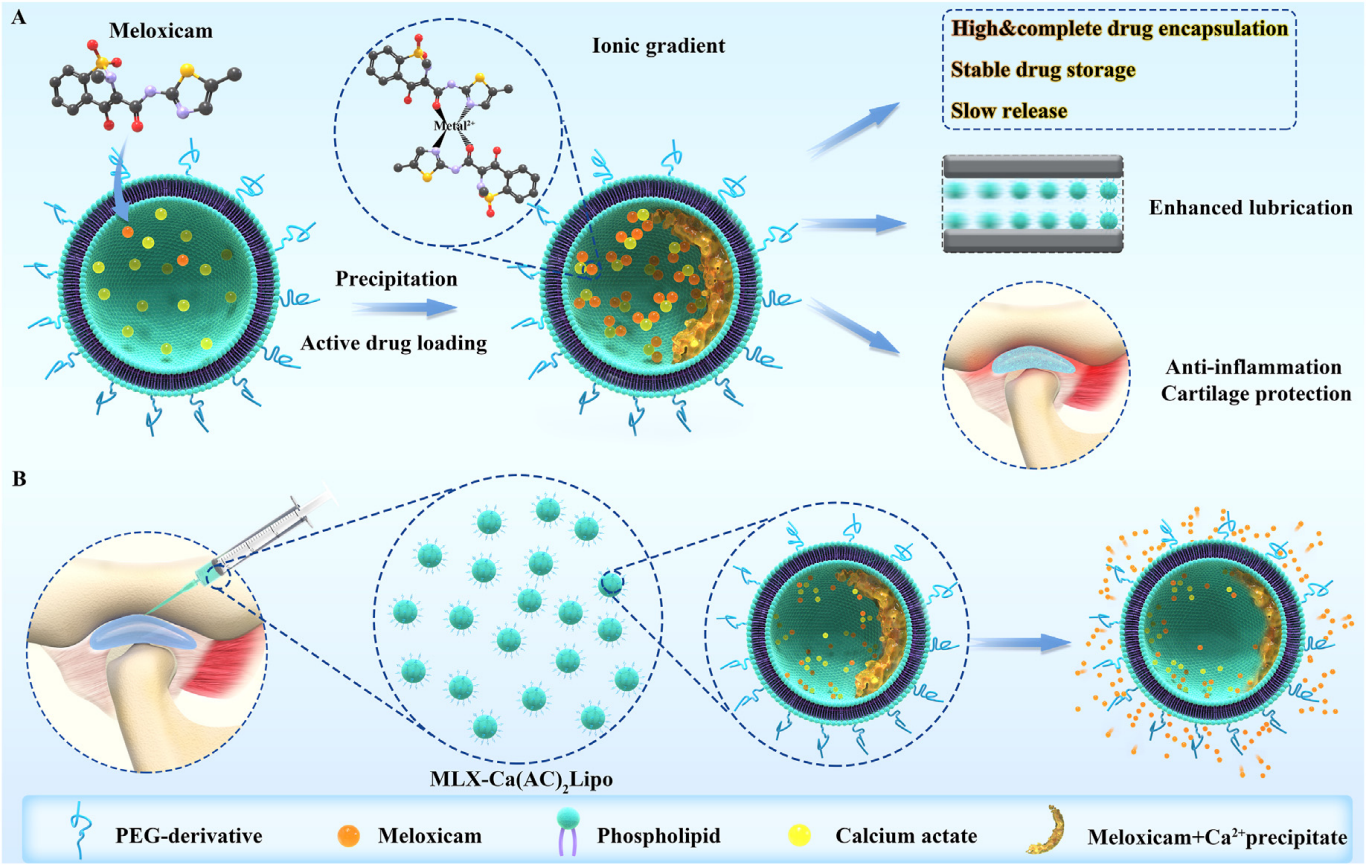
(A) Schematic illustration showing actively-loaded meloxicam liposomes (MLX-Ca(AC)_2_Lipo) and its main advantages (compared with passive loading method) such as high & complete drug encapsulation, stable drug storage, and slow drug release. (B) Schematic illustration showing local intra-articular injection of MLX-Ca(AC)_2_Lipo to treat rat TMJ osteoarthritis and the release mode of meloxicam from the system.

In this study, we developed novel meloxicam liposomes prepared by active loading method. Two major problems were solved for meloxicam's poor aqueous solubility and high lipophilicity by using meglumine to enhance aqueous solubility and divalent metal solution to improve encapsulation efficiency. Meloxicam was effectively entrapped by calcium ions (Ca^2+^) in the internal aqueous phase of liposomes as MLX-Ca(AC)_2_Lipo. Compared with passively-loaded meloxicam liposomes with a encapsulation efficiency of 60.6%, MLX-Ca(AC)_2_Lipo achieved an increased encapsulation efficiency as high as 98.4% along with a sustained drug release profile. The effect of MLX-Ca(AC)_2_Lipo was evaluated both *in vitro* and *in vivo* employing a rat TMJ osteoarthritis model via local intra-articular injection. It was anticipated that MLX-Ca(AC)_2_Lipo could not only effectively reduce chondrocytes apoptosis and extracellular matrix degeneration based on the mechanism of decreasing the expression of COX-2 but also greatly increase the hydration lubrication of the joint to inhibit cartilage degeneration, as shown in Scheme 1.

## 2 Materials and methods

### 2.1 Materials and reagents

Meloxicam (>99% purity) was purchased from TCI chemicals Co., Ltd. (Shanghai, China). Distearoyl phosphaethanolamine-polyethylene glycol 2000 (DSPE-PEG2000), hydrogenated soybean phosphatidylcholine (HSPC), and cholesterol were purchased from Nippon Fine Chemical Co., Ltd. (Osaka, Japan). Divalent metal salts (analytical grade) used in this study, including calcium acetate, zinc sulfate, magnesium sulfate, and manganese sulfate, and Dowex resin were purchased from Merck KGaA (Darmstadt, Germany). Live-Dead Staining Kit was purchased from Beyotime Biotech Co., Ltd. (Shanghai, China). Cell Counting Kit-8 was purchased from Dojindo Laboratories (Kyushu, Japan). Apoptosis Detection Kit was purchased from KGI Biotechnology Development Co., Ltd. (Shanghai, China). Purified water (18.2 ​MΩ ​cm resistivity at 25 ​°C) from Milli-Q® Direct System (Merck, Darmstadt, Germany) was employed for preparation of all the solutions.

### 2.2 Preparation of meloxicam liposomes

Actively-loaded meloxicam liposomes were prepared using divalent metal solutions as the “trapping agent”. Briefly, HSPC, DSPE-PEG2000, and cholesterol (3:1:1, w/w) were dissolved in anhydrous ethanol and then mixed with acetate or sulfate salts of Ca^2+^, Zn^2+^, Mn^2+^, and Mg^2+^ solutions with the concentration of 300 ​mM. The mixture was stirred at 60 ​°C for 30 ​min. The liposome suspension was sequentially extruded through 200, 100, 80, and 50 ​nm of polycarbonate membranes (Cytiva, Marlborough, MA, USA) in the Lipex extruder (Evonik Industries AG, Essen, Germany), and small unilamellar vesicles were obtained. The vesicles were dialyzed against 10% sucrose solution (w/v) overnight at 4 ​°C to remove the divalent metal ions outside the liposomes, forming the transmembrane gradient of metal ions for active drug loading. Meloxicam-meglumine complex solution (10 ​mg/mL) was added to the dialyzed liposomes and incubated at 60 ​°C for 30 ​min to result in the meloxicam concentration of around 1 ​mg/mL. After loading, the meloxicam liposomes were dialyzed against 10% sucrose solution (w/v) to eliminate the un-encapsulated meloxicam as well as meglumine.

For comparison, passively-loaded meloxicam liposomes were also prepared by thin film hydration method [28] using the same lipid composition. Lipids were dissolved in chloroform, and meloxicam powder at the drug-to-lipid ratio of 0.1 (molar ratio) was added in the solution. Chloroform was removed employing a rotary vacuum evaporator at 40 ​°C. The dried thin film was hydrated with Milli-Q water to obtain the meloxicam liposome suspension, which was further homogenized by a probe sonicator (Scientz-IID, Ningbo, China) at the power of 50 ​W. Probe sonication of the liposome suspension incubated in an ice-water bath was continuously performed for 30 ​s with a pause of 30 ​s, and this process was repeated for three times. The resulted liposomes were referred as MLX-WaterLipo.

### 2.3 Characterizations of meloxicam liposomes

#### 2.3.1 Encapsulation efficiency

The encapsulation efficiency (EE%) of the actively-loaded meloxicam liposomes was measured using the resin exchange method [29,30]. Briefly, meloxicam liposomes were diluted with saline and the total concentration of meloxicam (*C*_total_) was measured by ultraviolet (UV) method at *λ*_max_ ​= ​362 ​nm employing a microplate reader (M200PRO, TECAN, Switzerland), as shown in Fig. S1. Afterwards, Dowex resin was added to the diluted liposomes to adsorb the un-encapsulated meloxicam, and thus the concentration of the liposomal encapsulated meloxicam (*C*_inner_) could be measured. The encapsulation efficiency of the meloxicam liposomes was calculated by the following equation.

$$EE\left(\%\right)=\frac{C_{inner}}{C_{total}}\times 100\%$$

For the passively-loaded meloxicam liposomes, the values of *C*_total_ and *C*_inner_ were measured based on the UV absorbance of the ethanol-diluted liposomes before and after dowex resin input (ethanol/liposome ​= ​5:1, v/v). Finally, the encapsulation efficiency of the passively-loaded meloxicam liposomes was calculated by the above equation and compared with that of the actively-loaded meloxicam liposomes.

#### 2.3.2 Particle size and liposome morphology

Actively-loaded meloxicam liposomes using calcium acetate as the transmembrane gradient with the highest encapsulation efficiency were selected for further study. The corresponding liposomes were referred as MLX-Ca(AC)_2_Lipo hereinafter. The particle size of MLX-Ca(AC)_2_Lipo and MLX-WaterLipo was measured using the dynamic light scattering method (Nanosizer ZS90, Malvern, UK) at 25 ​°C. The liposomes were diluted with saline to the concentration of 50 ​μg/mL, and the measurement of particle size was performed triplicate. The direct imaging of the liposomes was achieved employing a cryo-transmission electron microscope (TEM, FEI Talos G2, ThermoFisher Scientific, Waltham, MA, USA). Vitrified specimens were prepared on a copper grid coated with 300 mesh of perforated lacey carbon (Ted Pella, Redding, CA, USA). Prior to specimen preparation, the grid was plasma-etched to increase its hydrophilicity. Typically, a drop of the solution was applied to the grid in a controlled environment (25 ​°C and 90∼100% relative humidity), and blotted with a filter paper to form a thin liquid film of solution. The automatically blotted sample was immediately plunged into liquid ethane (Vitrobot Mark IV, ThermoFisher Scientific, Waltham, MA, USA). The samples were examined at 120 ​kV in low-dose mode to minimize radiation damage from the electron beam.

#### 2.3.3 Thermodynamic characterization

The thermodynamic performance of MLX-Ca(AC)_2_Lipo and MLX-WaterLipo was evaluated by differential scanning calorimetry (DSC). The liposomes were diluted with 10% sucrose solution (w/v) to the lipid concentration of 8 ​mg/mL. The samples were injected to NanoDSC (TA Instruments, New Castle, DE, USA) and scanned at the rate of 1 ​°C/min in the temperature range of 5∼95 ​°C using 10% sucrose solution (w/v) as the reference. The thermodynamic data were analyzed using the NanoAnalyzer software (TA Instruments, New Castle, DE, USA) after baseline correction and normalization with corresponding drug or lipid concentrations. The thermodynamic behavior of blank (drug-free) calcium acetate liposomes was also obtained for comparison.

#### 2.3.4 In vitro drug release behavior

The *in vitro* drug release behavior of MLX-Ca(AC)_2_Lipo and MLX-WaterLipo was evaluated by the “reverse diffusion” method [31,32] employing a home-made dialysis device, which was prepared by replacing the polycarbonate membrane (pore size: 0.4 ​μm) in the 12-well Corning® Transwell insert (Merck, Darmstadt, Germany) with even smaller pore size (50 ​nm). Afterwards, the liposome solution (1.2 ​mL) was added to the lower chamber (receiver chamber), and phosphate buffered saline (PBS, 100 ​mM, pH ​= ​7.2–7.4) or fresh synovial fluid collected from temporomandibular disorder (TMD) patients in the volume of 0.4 ​mL was put to the upper chamber (the insert). The dialysis device was incubated in a temperature-controlled shaker at 37 ​°C and a shaking speed of 50 round per minute (rpm). At the time intervals of 0, 0.25, 0.5, 1, 2, 4, 18, 24 ​h, and afterwards, every 24 ​h till 168 ​h (7 days), 0.2 ​mL of the release medium in the upper chamber was removed and further refilled with 0.2 ​mL of fresh release medium. The UV absorbance of the samples at 362 ​nm was measured using the microplate reader. The cumulative drug release of meloxicam liposomes was calculated by the following equation, where *C*_*n*_ was the concentration of the sample taken at the *n*th time point, and *W* was the total amount of meloxicam added to the lower chamber. In addition, 1.6 was the total volume of the liquid in the dialysis device.

$$Cumulative\;drug\;release\left(\%\right)=\frac{\sum_{i=0}^{n}{1.6C}_{n}}{W}\times 100\%$$

In order to evaluate the effect of the dialysis device on the release rate of meloxicam, 1.2 ​mL of meloxicam-meglumine solution (1 ​mg/mL) was put to the dialysis device, and the release behavior was examined using the same method described above.

#### 2.3.5 Storage stability

The short term storage stability of MLX-Ca(AC)_2_Lipo and MLX-WaterLipo was examined by measuring the encapsulation efficiency of the liposomes after storage for 7 days at 4 ​°C and then compared with that right after preparation. Additionally, the long term stability of MLX-Ca(AC)_2_Lipo was further investigated under the same storage conditions by measuring encapsulation efficiency and particle size at the time intervals of 0, 10, 20, 30, and 60 days, respectively.

#### 2.3.6 Lubrication property

The lubrication property of MLX-Ca(AC)_2_Lipo was evaluated employing an atomic force microscope (AFM, MFP-3D, Asylum Research, USA) under the contact mode according to our previous method [33,34]. A polystyrene microsphere with a diameter of 5 ​μm was attached to the tip of the AFM probe by UV irradiation for 40 ​min. Silicon wafer (Electron Microscopy Sciences, Hatfield, PA, USA) was thoroughly cleaned with acetone-ethanol solution and then put in a 24-well cell culture plate (Corning®, Merck, Darmstadt, Germany), which was incubated with the meloxicam liposomes at different concentrations (i.e., 2, 5, and 10 ​mM) at 4 ​°C for 1 ​h. Afterwards, the silicon wafer was rinsed with saline to remove the un-adsorbed liposomes and fixed in the sample stage. A transverse scanning was performed in the area of 20 ​μm ​× ​5 ​μm ​at the frequency of 2 ​Hz. The friction force between the polystyrene microsphere at the tip of the probe and the liposome-adsorbed silicon surface was recorded. The elastic coefficient of the probe cantilever used in the study was 0.1 ​N/M. The applied load was adjusted in the scanning process, which were 20, 40, 60, 80, 100, and 120 ​nN, respectively. The coefficient of friction (COF), which was defined as the ratio of the friction force to the probe applied load, was calculated based on the Asylum Research software.

### 2.4 Biocompatibility

The cells used in the following *in vitro* test were ATDC5 mouse chondrogenic cell line, which were purchased from Shanghai National Collection of Authenticated Cell Cultures. The cells were placed in an incubator to proliferate at 37 ​°C and 5% CO_2_. The cells were cultured in the DMEM/F12 medium (Gibco BRL, Gaithersburg, MD, USA), which was supplemented with 10% fetal bovine serum (Biological Industries, USA) and 1% antibiotic/antimycotic (Invitrogen, USA). The culture medium was refreshed every 2–3 days. The cells were removed to be passaged at a ratio of 1:3 when 70–80% confluency was reached. Additionally, in this study Ca^2+^ was selected for active loading of meloxicam in the liposomes. To ensure that Ca^2+^ introduced with the intra-articular injection of MLX-Ca(AC)_2_Lip would not lead to the occurrence of precipitate, calcium acetate (300 ​mM, pH ​= ​6.5) was added to fresh synovial fluid from TMD patients at a Ca^2+^ concentration of 6 ​mM. The turbidity of Ca^2+^-synovial fluid mixture solution was evaluated by measuring the UV absorbance at 600 ​nm using the fresh synovial fluid as the comparison.

#### 2.4.1 Cell Counting Kit-8 (CCK-8) assay

The ATDC5 mouse chondrogenic cell line was digested, counted, and cultured for 12 ​h to adhere to the dishes. DMEM high-glucose medium with 10% fetal bovine serum containing 0, 2, 20, 50, and 100 ​μM of MLX-Ca(AC)_2_Lipo was exchanged to cultured cells, respectively. The observation period was every 48 ​h for a duration of 7 days, and 5 replicate wells were set in each group. 10 ​μL of CCK-8 detection solution was added to each well of the plate and further incubated at 37 ​°C in the dark for 2 ​h. The optical density (OD) value of each well was measured at 450 ​nm with the microplate reader to examine the effect of various concentrations (i.e., 2, 20, 50, 100, and 200 ​μM) of MLX-Ca(AC)_2_Lipo on cell proliferation.

#### 2.4.2 Live-Dead staining

ATDC5 mouse chondrogenic cell line was co-cultured with different concentrations of MLX-Ca(AC)_2_Lipo (i.e., 2, 20, 50, 100, and 200 ​μM) and the DMEM high-glucose medium containing 10% fetal bovine serum for 24 ​h, and then Live-Dead cell staining was performed. The live and dead cells were labeled with Calcein-AM and propidium iodide (PI), respectively. Under fluorescence microscope, the live and dead cells could be observed releasing green and red fluorescence. Based on this method, the toxicity and proliferation ability of the cells co-cultured with different concentrations of MLX-Ca(AC)_2_Lipo could be investigated.

#### 2.4.3 Flow cytometry

ATDC5 mouse chondrogenic cell line was co-cultured with the above-mentioned different concentrations of MLX-Ca(AC)_2_Lipo and the DMEM high-glucose medium containing 10% fetal bovine serum for 24 ​h. Afterwards, the cells and cell supernatants were collected with trypsin digestion solution without ethylene diamine tetraacetie acid. After centrifugation at the speed of 1000 ​rpm for 5 ​min, the supernatant was discarded and the cells were rinsed with PBS. The cells were further centrifuged at 1000 ​rpm for 5 ​min, and 10 ​μL of Annexin V-FITC binding solution was added to gently resuspend the cells. Subsequently, 10 ​μL of PI staining solution was added and mixed gently. The solution was incubated at room temperature (20-25 ​°C) for 10–20 ​min in the dark, which was then placed on ice. The proportion of apoptotic cells was determined by using flow cytometry detection (CytoFLEX, Beckman Coulter, USA).

### 2.5 In vitro anti-inflammatory effect

#### 2.5.1 Real-time quantitative polymerase chain reaction

ATDC5 mouse chondrogenic cell line was seeded in 12-well plate and cultured for 12 ​h until the cell density was confluent to about 70%–80%. Afterwards, the cells were co-cultured with the tumor necrosis factor (TNF-α) for 24 ​h, and 0, 2, 20, and 80 ​μM of MLX-Ca(AC)_2_Lipo was added for intervention for another 24 ​h. The total RNA of the cells after treatment was obtained by RNA Express Total RNA Kit (Ncmbio, Suzhou, China). The NanoDrop 2000/2000C spectrophotometer was used to examine the purity and concentration of RNA at the wavelength of 260/280 ​nm. The Hifair® Ⅱ 1st Strand cDNA Synthesis Kit (Yeasen, China) was used to reverse transcribe 1 ​μg of extracted RNA into complementary DNA (cDNA). The resultant cDNA was used as the template in the Hieff UNICON Universal Blue qPCR SYBR Green Master Mix (Yeasen, China) and real-time quantitative polymerase chain reaction (RT-qPCR). The reactions were performed on a Light Cycler96 Real-Time PCR System (Roche, Basel, Switzerland). The primer sequences used in this study were displayed in Table 1. The expression level of mRNA including matrix metalloproteinases-3 (MMP3), matrix metalloproteinases-13 (MMP13), a disintegrin and metalloproteinase with thrombospondin 4 (ADAMTS4), COX-2, and glyceraldehyde-3-phosphate dehydrogenase (GAPDH) was determined using specific primers by the 2^−ΔΔCt^ method and normalized to GAPDH.

**Table 1:** List of RT-qPCR primer sequences in this study.

Gene name		5′-3′ sequence (forward; reverse)
MMP3	Forward	5′- CAGTCCCTCTATGGAACTCCC-3′
	Reverse	5′- AGGGTGCTGACTGCATCAAA-3′
MMP13	Forward	5′- GACCCCAACCCTAAGCATCC-3′
	Reverse	5′- CAGGCGCCAGAAGAATCTGT-3′
ADAMTS4	Forward	5′- CAAGCATCCGAAACCCTGTC-3′
	Reverse	5′- ACACAGGTCCTGCCGGG-3′
COX-2	Forward	5′- GCAGGAAGTCTTTGGTCTGG-3′
	Reverse	5′- AGTTGCTCATCACCCCACTC-3′
GAPDH	Forward	5′- AACTTTGGCATTGTGGAAGG-3′
	Reverse	5′- ACACATTGGGGTAGGAACA -3′

Similarly, ATDC5 mouse chondrogenic cell line was cultured based on the above-mentioned conditions. RNA was extracted after the cells were treated with 10 ​ng/mL of TNF-α or 10 ​ng/mL of TNF-α + 20 ​μM of MLX-Ca(AC)_2_Lipo for 0, 1, 3, 6, 12, and 24 ​h, respectively. The mRNA expression levels of MMP3, MMP13, ADAMTS4, and COX-2 were examined at different time points.

#### 2.5.2 Western blot

ATDC5 mouse chondrogenic cell line was seeded in 6-well plate and cultured for 12 ​h until the cell density was confluent to about 70%–80%. After that the cells were co-cultured with TNF-α for 24 ​h, and then 20 ​μM of MLX-Ca(AC)_2_Lipo was added for incubation for another 24 ​h. The protein lysis buffer was used to collect the total protein of the cells. The samples were homogenized in protein lysis solution (supplemented with 1% phenylmethanesulfonyl fluoride and 2% phosphatase inhibitor), lysed for 30 ​min on ice, and centrifuged at 12,000 ​rpm for 15 ​min at 4 ​°C. Both the supernatant and the total protein content were collected and determined by Bicinchoninic Acid assay. The same concentration of protein (20 μg/lane) was loaded on a sodium dodecyl sulfate-polyacrylamide gel electrophoresis (SDS-PAGE) gel. After proteins were transferred to the 0.45 ​μm of poly(vinylidene fluoride) membrane, the membrane was blocked with 5% bovine serum albumin (BSA) at room temperature for 1 ​h and then incubated with primary antibody overnight at 4 ​°C (MMP3, absin, 1:1000; MMP13, proteintech, 1:1000; ADAMTS4, abcam, 1:1000; COX-2, abcam, 1:1000; GAPDH, Huaxingbio, 1:3000). Afterwards, the poly(vinylidene fluoride) membrane was washed using Tris-HCl and Tween 20 buffer for 5 ​min and three times, then it was co-incubated with the secondary antibodies for 1 ​h at room temperature. The protein bands were visualized using a Bio-rad Gel Doc XR ​+ ​image scanning system (Bio-rad. Inc., CA, USA).

#### 2.5.3 Immunofluorescence staining

ATDC5 mouse chondrogenic cell line was co-cultured with the above-mentioned conditions. The cells were fixed with 4% paraformaldehyde for 15 ​min, permeated in 0.5% Triton X-100 at room temperature for 15 ​min, and blocked with 5% BSA for 30 ​min. The cell sheets were incubated with primary antibody overnight at 4 ​°C (MMP3, 1:200; ADAMTS4, 1:200). On the second day, the cell sheets were co-incubated with rhodamine-labeled secondary antibody and Fluorescein isothiocyanate-labeled (FITC) phalloidin for 1 ​h at 37 ​°C. The samples were stained with 4, 6-diamidino-2-phenyindole dilactate (DAPI), and the images were observed by a confocal fluorescence microscope (LSM800, ZEISS, Germany).

### 2.6 In vivo therapeutic outcome

A total of eighteen female Sprague-Dawley rats (weight: 200–250 ​g; 8-week-old) were provided by Shanghai JieSiJie Laboratory Animal Co., Ltd. and then housed in the pathogen-free room. All the rats were fed with sterilized food and redistilled water. The protocols of *in vivo* animal experiments were approved by the Ethics Committee of Ninth People's Hospital, Shanghai Jiao Tong University School of Medicine (SH9H-2021-T141-1). A unilateral anterior crossbite (UAC) model was adopted to establish TMJ osteoarthritis model based on previously reported studies [35,36]. The rats were randomized into three groups (six rats for one group), which were received different treatments (Control, UAC ​+ ​PBS intra-articular injection, and UAC ​+ ​unilateral MLX-Ca(AC)_2_Lipo intra-articular injection), respectively. The rats were anesthetized by the intraperitoneal injection of 1% pentobarbital sodium on the 2nd, 3rd, 4th, and 5th weeks after the UAC model was established. After anesthesia, the needle of insulin syringe (1 ​mL) was inserted under the left zygomatic arch until it reached the mandibular ramus's outer surface. The direction of the needle was adjusted to travel along the bone surface and eventually reached the TMJ supra-articular space. The procedure was performed with great care in order not to damage the surrounding maxillofacial muscles, nerves, and glandular tissues. Subsequently, 20 ​μL (4 ​mM) of MLX-Ca(AC)_2_Lipo or the same volume of PBS was locally injected into the TMJ supra-articular space of the rats in the UAC ​+ ​MLX-Ca(AC)_2_Lipo group or UAC ​+ ​PBS group. The intra-articular injections were given once every 7 days for consecutively four weeks until the end of experiment. All the rats were euthanized by intraperitoneal injection with excessive pentobarbital sodium. The left TMJ samples of all the rats were prepared for sections with a thickness of 6 ​μm. The sections were deparaffinized in xylene and dehydrated by a graded series of ethanol solutions. Then the sections were stained alternately by hematoxylin-eosin (HE) and 0.4% Safranin-O solution and 0.1% fast green solution (Sigma-Aldrich, USA) for immunohistochemical analysis. The stained sections were observed by an optical microscope, and the images were captured using the Leica DFC490 system. The images included typical fibrous layer, proliferation cell layer, hypertrophic cartilage layer, and endochondral osteogenic layer. Furthermore, condylar cartilage destruction was scored by three independent observers blinded to the experimental information employing the Osteoarthritis Research Society International (OARSI) grading system reported in the literature [37,38]. The ImageJ software (NIH, USA) was used for image analysis.

### 2.7 Statistical analysis

All statistical analyses were performed using the SPSS software (version 23.0, IBM Corp, Armonk, NY, USA). The data were displayed as mean value ​± ​standard deviation (SD). One-way analysis of variance (ANOVA) was used for the evaluation of the data of cell proliferation in CCK-8 assay, cell counting in Live-Dead staining, and live cells rate in flow cytometry for the meloxicam liposomes. An unpaired *t*-test was used for the analysis and comparison of the *in vitro* and *in vivo* experimental results between different groups. The statistical difference was considered significant when p ​< ​0.05.

## 3 Results and discussion

### 3.1 Development of actively-loaded meloxicam liposomes

**Fig. 1:**
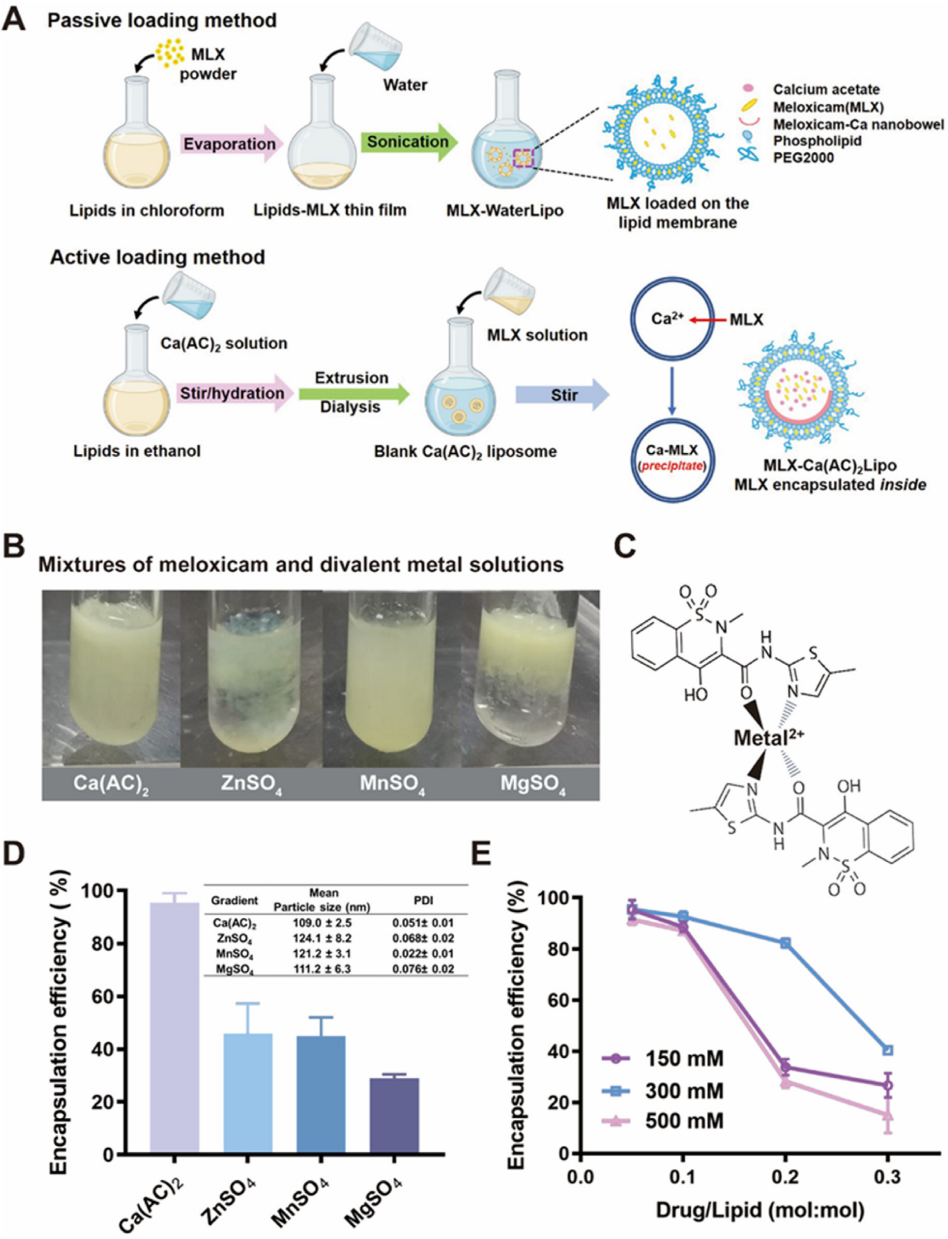
Development of actively-loaded meloxicam liposomes. (A) Illustration showing the passive and active drug loading method for the preparation of meloxicam liposomes. (B–E) Effect of the type and concentration of divalent metal ions in the intra-liposomal aqueous phase on the encapsulation efficiency of actively-loaded meloxicam liposomes. (B) Meloxicam precipitations in calcium acetate, zinc sulfate, manganese sulfate, and magnesium sulfate solutions (300 ​mM, pH ​= ​6.5–7.4). (C) Meloxicam-divalent metal precipitates are proposed to be formed via electrostatic interaction between the negative charge of enol ionization of meloxicam and the metal cations under neutral environment. (D) Encapsulation efficiency and particle size of actively-loaded meloxicam liposomes using the four divalent metal solutions as the internal aqueous phase (300 ​mM, pH ​= ​7) at the drug-to-lipid ratio of 0.1. (E) Effect of the concentration of calcium acetate in the internal aqueous phase on the encapsulation efficiency of actively-loaded meloxicam liposomes.

Liposomal drug loading methods are generally classified as passive or active based on different mechanisms, as shown in Fig. 1A. As a hydrophobic drug, meloxicam is commonly loaded in liposomes via passive loading method, where the drug is typically incorporated in liposomal membrane co-currently with liposome formation [39,40]. Passive drug loading usually reduces liposome stability and is much less efficient than active drug loading. For passively-loaded meloxicam liposomes, the relatively low drug concentration, poor storage stability, and burst release greatly restrict the application of intra-articular injection. In contrast, active drug loading can be more efficient, resulting in high intra-liposomal concentration and good storage stability by drug encapsulation inside the liposomes [41]. Active drug loading is achieved by transmembrane gradients. The amphiphilic drugs dissolved in the exterior phase of the liposomes permeate across the lipid membrane to the interior phase and are encapsulated by the “trapping agent” to complete the drug loading process.

According to the Biopharmaceutical Classification System, meloxicam is a typical type II drug and has a saturated water solubility as low as 7.15 ​mg/L (25 ​°C) and a high partition coefficient of 3.54 [42]. Consequently, a very limited amount of meloxicam can dissolve in water and be encapsulated inside the liposomes. Meloxicam is highly soluble in basic solutions such as NaOH at the pH value of 8 and above. However, lipid degradation is accelerated at high pH values under the loading temperate of 60 ​°C [43]. In order to solve this problem, we selected meglumine to improve the water solubility of meloxicam at *neutral* pH in the present study. It is reported that meloxicam and meglumine can form salt *in situ* during melt extrusion, after which the water solubility and chemical stability of meloxicam is significantly improved [44]. In this study, the meloxicam-meglumine salt is prepared by mixing the two compounds at different molar ratios in methanol and stirred overnight at room temperature. Yellow powder product is collected following rotary evaporation of methanol at 40 ​°C. The DSC and powder X-ray diffraction (XRD) characterization results as shown in Fig. S2 show that the meloxicam-meglumine salt is in amorphous state at the molar ratio of 1:1, and it dramatically increases the water solubility of meloxicam to around 20,000 ​mg/L (pH ​= ​7). Consequently, it provides a high concentration of the unionized permeable species as the driving force for active loading of meloxicam. Based on the high hydrophilicity and ionizable property of meglumine, it is considered that meglumine will not permeate along with meloxicam to the internal space of the liposomes in the drug loading process. Instead, the dissociated meloxicam will enter the liposomes and be trapped inside.

The next is to find an intra-liposomal “trapping agent” that has a strong interaction with meloxicam to compete against its membrane partition for efficient active loading. It is noted in the experiment that yellow to light-yellow precipitates are formed when meloxicam-meglumine complex is mixed with divalent metal solutions including Ca^2+^, Zn^2+^, Mn^2+^, and Mg^2+^ (Fig. 1B), which is possibly attributed to electrostatic interaction between the negatively charged meloxicam and cationic metal ions (Fig. 1C). However, the mixtures maintain transparent when the meglumine solution is mixed with the four divalent metal solutions (Fig. S3). Based on these results, the above four divalent metal solutions are used to prepare blank liposomes for active loading of meloxicam, and then the encapsulation efficiency of the liposomes is examined. As demonstrated in Fig. 1D, Ca^2+^ presents the strongest capacity for active loading of meloxicam among the four divalent metals with an encapsulation efficiency higher than 90%. However, the other three metal solutions only results in encapsulation efficiencies of less than 50%. The four kind of meloxicam liposomes actively-loaded by divalent metal ions are in the size range of 110∼125 ​nm with a narrow distribution of polydispersity index (PDI, ranging from 0.02 to 0.08).

As discussed above, the encapsulation efficiency of the actively-loaded meloxicam liposomes using the divalent cations as the “trap agents” is mainly determined by three parameters including (1) the binding capacity of divalent cations to meloxicam to form precipitates, (2) the concentration of divalent cations inside the liposomes, and (3) the driving force for continuous active drug loading (i.e., continuous drug permeation from outside to inside of liposomes and be trapped). All the four divalent cations can bind to meloxicam and result in the formation of precipitates. However, Zn^2+^ is reported to be permeable across the lipid membrane, and it can also cause the bilayers to swell [45,46]. Therefore, Zn^2+^ inside the liposomes will probably leak out during dialysis process before meloxicam loading, resulting in reduced encapsulation efficiency. Meloxicam is proposed to bind with the divalent cations through electrostatic interaction. There are two dissociation constant values for meloxicam (pKa1 ​= ​1.1, pKa2 ​= ​4.7). Therefore, it is critical to keep neutral or slightly basic pH value inside the liposomes, under which meloxicam is negatively charged for active loading. For Ca(AC)_2_ gradient, the efflux of acetic acid as one highly permeable molecule across the liposomal bilayer membrane (6.6 ​× ​10^−4^ ​cm/s [47]) can behave as the proton shuttles to establish the transmembrane gradient for loading of weak amphiphilic acid molecules. That is to say, the efflux of acetic acid will promote influx of meloxicam, and the exchange between two molecules may serve as a “chemical engine” for continuous meloxicam loading [48]. Differently, sulfate ion is very hard to permeate across lipid membrane (permeability <10^−12^ ​cm/s [49]). Therefore, for the liposomes containing sulfatesalts of Mg^2+^ and Mn^2+^, local pH inside the liposomes will decrease with meloxicam influx because meloxicam donates H^+^ and becomes negatively charged. When the internal pH value of liposomes approaches the dissociation constant of meloxicam, few negatively charged molecules are available for binding with the divalent metal ions and thus the drug encapsulation will be stopped. To confirm the above anticipation, we prepared meloxicam liposomes with CaCl_2_ (300 ​mM), instead of Ca(AC)_2_, at the same concentration of Ca^2+^, and measured the encapsulation efficiency. Chloride is less permeable (reported permeability of 3 ​× ​10^−8^∼2 ​× ​10^−9^ ​cm/s [50]) than acetate, therefore it cannot exchange with meloxicam. As expected, the liposomes using CaCl_2_ as “trapping agent” result in an encapsulation efficiency of about 41.6% (data not shown), which is comparable to that prepared with MnSO_4_ and MgSO_4_ solutions.

Calcium acetate is used as the loading gradient and its concentration is optimized for active loading of meloxicam. As displayed in Fig. 1E, at all the three drug-to-lipid ratios (0.1, 0.2, and 0.3), meloxicam liposomes prepared by 300 ​mM of calcium acetate achieves the highest encapsulation efficiency (>90%, 83.5%, and 40.7%, respectively) among the three groups (150, 300, and 500 ​mM). Therefore, 300 ​mM of calcium acetate solution has been selected for the preparation of actively-loaded meloxicam liposomes, and the drug-to-lipid ratio is set at 0.1 in the following test. The meloxicam liposomes are referred as MLX-Ca(AC)_2_Lipo and used in the *in vitro* and *in vivo* studies.

### 3.2 Characterizations of meloxicam liposomes

The actively-loaded meloxicam liposomes (MLX-Ca(AC)_2_Lipo) were smaller and more uniform than passively-loaded meloxicam liposomes (MLX-WaterLipo), which is described by the average diameter of 110 ​nm vs 228.4 ​nm and PDI of 0.051 vs 0.477. The size difference is attributed to the homogenization methods used in the preparation of the two kinds of meloxicam liposomes. The blank Ca(AC)_2_ liposomes for the active loading of meloxicam are extruded through the polycarbonate membranes (size: 200, 100, and 80 ​nm) employing a Lipex extruder (Northern Lipids, Canada), resulting in a smaller particle size and narrow distribution. However, MLX-WaterLipo prepared by thin film method is homogenized using a probe sonicator, thus the particle size is much larger with a relatively wide distribution. In addition, MLX-Ca(AC)_2_Lipo shows a high encapsulation efficiency of 98.4%, which is significantly higher than that of MLX-WaterLipo (60.6%). The concentrations of meloxicam in these two kinds of liposomes are 0.98 and 0.61 ​mg/mL. Generally, it is more difficult to achieve a high drug to lipid molar ratio for poorly water soluble drugs compared to water soluble drugs. In one study of active loading of a poorly soluble drug (AR-67) in liposomes based on calcium acetate gradient, [51], a supersaturated drug solution and a lipid concentration as high as 67mM were applied to increase encapsulation efficiency. The highest drug to lipid molar ratio was reported as0.17 ​with drug concentration of 0.6 ​mg/mL. In our study, the actively-loaded meloxicam liposomes successfully achieved a drug to lipid molar ratio of 0.1, and the drug concentration is around 1 ​mg/mL with 20 ​mM of lipids.

**Fig. 2:**
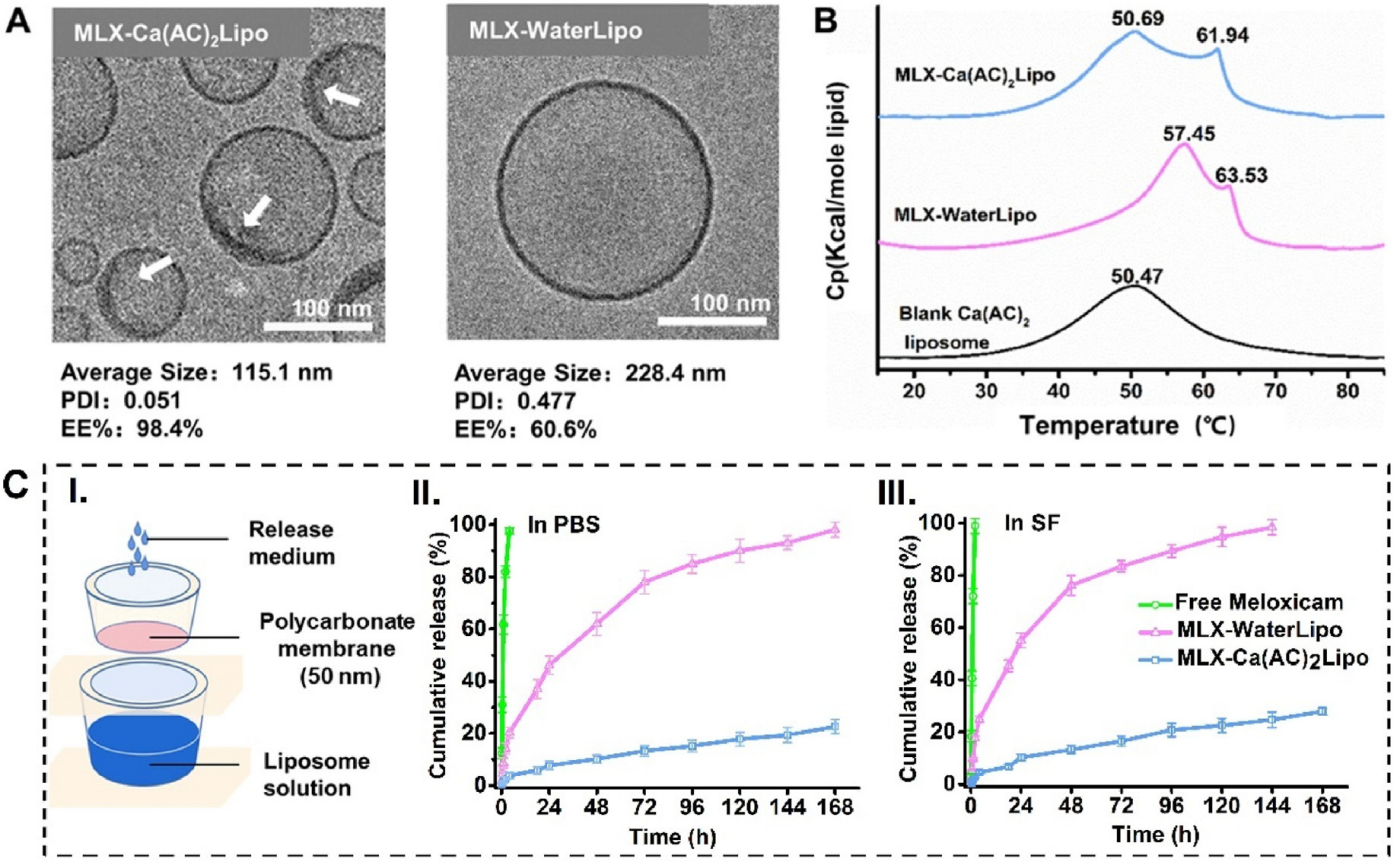
Comparison of meloxicam liposomes prepared by the active or passive loading method. (A) Cryo-TEM morphology of two kinds of liposomes, where the white arrows indicate the presence of nano-bowl structure in the inner surface of the actively-loaded liposomes. (B) DSC thermograms of two kinds of liposomes and blank calcium acetate liposome. (C) *In vitro* drug release behavior of two kinds of meloxicam liposomes over 7 days. (I.) Schematic diagram showing a home-made dialysis device; (II.) Cumulative drug release curves in PBS and (III.) in fresh synovial fluid (SF) from TMD patients.

Under cryo-TEM, these two kinds of meloxicam liposomes are single unilamellar vesicles with the diameter of about 100 and 200 ​nm, respectively, as shown in Fig. 2A and Fig. S4. Specifically, a typical nano-bowl shaped structure is observed in the inner wall of MLX-Ca(AC)_2_Lipo (indicated by the white arrows in Fig. 2A), which is similar to the result of doxorubicin liposome reported in a previous study [52]. However, the nano-bowls observed were firstsynthesized by silica then coated with lipids to form liposomes before encapsulation of doxorubicin Differently, in our study the nano-bowl structure is formed simultaneously during the process of actively loading of meloxicam in the liposomes. This phenomenon is proposed to be related with the “fixed” calcium ions at the inner surface of the bilayer membrane by the negatively charged headgroups of phospholipids. The calcium ions can further attract and bind to the negatively charged meloxicam in the internal aqueous phase, leading to the formation of nano-bowl shaped meloxicam-calcium precipitates closely attached to the inner surface of the liposomal membrane. It is interesting to observe that the nano-bowel structure covers only a part of the inner surface of liposomes. A similar phenomenon has been reported in previous study, where a poorly water soluble drug ( all-trans-retinoic acid) is actively loaded with calcium acetate gradient [53]. It is suggested that incorporation of hydrophobic drugs to the lipid bilayer can change its original ordered structure and fluidize the membrane [54]. Considering the hydrophobicity of meloxicam, we think “partial covering ” of the meloxicam-calcium nano-bowel structure along the inner surface of liposomes may be attributed to meloxicam partition to the lipid bilayer that inhibits further interaction of calcium to the phospholipids.

The ordered and tenacious membrane structure of lipid formulation is crucial for preventing drug leakage or burst release. However, the interaction between meloxicam, a highly lipophilic drug, and membrane lipids can disturb the original ordered structure and affect drug release rate [55]. To investigate the meloxicam-membrane interaction in MLX-Ca(AC)_2_Lipo and MLX-WaterLipo, the thermal behavior of the liposomes is evaluated by measuring typical thermodynamic parameters including phase transition temperature (T_m_), enthalpy (ΔH), and cooperativity (ΔT_1/2_), which have been described in a previous study [56]. The blank liposomes (Fig. 2B, black curve) show one broad endotherm at the T_m_ of 50.47 ​°C with low enthalpy, which is in good consistence with the reported phase transition of phospholipid bilayer in ordered structure [57]. MLX-Ca(AC)_2_Lipo indicates two endotherms (Fig. 2B, blue curve). The first endotherm at 50.69 ​°C can be attributed to the membrane phase transition, and the second at 61.94 ​°C is probably caused by melting of meloxicam-calcium precipitates inside the liposomes (Table S1 and Fig. S5). The thermogram of MLX-Ca(AC)_2_Lipo indicates that the most meloxicam has been successfully encapsulated inside the liposomes with negligible interaction with the lipids. Simultaneously, MLX-Ca(AC)_2_Lipo maintains the same membrane structure as that of the blank liposomes. Both features are very favorable for achieving sustained drug release and excellent storage stability of the liposomes. MLX-WaterLipo also displays two endotherms with the major one up-shifted to 57.45 ​°C, and a tiny “shoulder” at 63.53 ​°C (Fig. 2B, pink curve). The fitting result demonstrates that membrane-incorporated meloxicam has significantly changed the bilayer structure of the liposomes, and thus burst drug release or fast drug leakage can occur. More detailed description and explanation of the DSC characterizations for the meloxicam liposomes are provided in the Supporting Information.

The *in vitro* drug release profiles of the two kinds of meloxicam liposomes are in good consistence with DSC results, as shown in Fig. 2C. In order to mimic the situation of meloxicam liposomes injected into the TMJ of rats, we used “reverse diffusion” method in the *in vitro* drug release test with a home-made dialysis device as illustrated in Fig. 2C–I. Different from the common “diffusion” method, in the "reverse diffusion" method, the liposomes are placed “outside” of dialysis bag/membrane, and the release samples are taken from the interior for the measurement of the released free drug permeated inside the dialysis bag. In this study, 1.2 ​mL of liposome solution is added to the lower chamber of the home-made release device, and 0.4 ​mL of PBS or fresh synovial fluid from the TMD patients is put to the upper (insert) chamber. Under such conditions, the liposomes are diluted about 1.3-fold, which is close to the dilution ratio following intra-articular injection of the liposomes into the TMJ of rats (the injected volume of liposomes is 20 ​μL, and the total joint cavity is assumed as 25 ​μL). This method can provide predictive information about the liposomal drug release in the TMJ of rats at a small dilution factor. In addition, the withdrawal of meloxicam from the upper chamber also well mimics elimination of the released drug in the joint. The time point has been recorded at which complete drug release for either meloxicam liposomes is achieved as the endpoint of the *in vitro* drug release test, since the result can well reflect the difference in release kinetics for the two kinds of meloxicam liposomes.

As shown from the *in vitro* drug release profiles of the meloxicam liposomes in both Fig. 2C–II (release medium: PBS) and Fig. 2C–III (release medium: fresh synovial fluid from TMD patients), MLX-WaterLipo (pink curve) exhibits a much faster drug release than MLX-Ca(AC)_2_Lipo (blue curve). Specifically, at 24 ​h, about 50% of meloxicam has been released from MLX-WaterLipo (46.1% in PBS and 55.2% in synovial fluid). The remaining meloxicam in MLX-WaterLipo shows a slow release until 168 ​h (in PBS) and 144 ​h (in synovial fluid) following an exponential profile that reflects a membrane-controlled passive diffusion process. In contrast, a linear sustained drug release profile is observed for MLX-Ca(AC)_2_Lipo, following zero-order kinetics in both PBS (R^2^ ​= ​0.97) and synovial fluid (R^2^ ​= ​0.96). At the endpoints of the *in vitro* drug release test in PBS (168 ​h) and in synovial fluid (144 ​h), less than 30% of meloxicam has been released from MLX-Ca(AC)_2_Lipo (22.6% and 27.9%, respectively). The result indicates that the drug release of MLX-Ca(AC)_2_Lipo is regulated by the disassociation of meloxicam-calcium precipitates inside the liposomes instead of simple diffusion of meloxicam across the lipid membrane. As the control group, almost all of the drug has been released from the meloxicam solution at 4 ​h in PBS and 2 ​h in synovial fluid (Fig. 2C–II and **2C-III**, green curve). It confirms that the polycarbonate membrane will not hinder the diffusion of meloxicam from the lower chamber to the release medium in the upper chamber. It is noted that a relatively faster drug release is observed for meloxicam liposomes and meloxicam solution in synovial fluid than those in PBS. It may be related to the high protein binding property of meloxicam [57]. Synovial fluid is an ultrafiltrate of plasma that originates from a highly fenestrated capillary network overlying synovial membrane. Synovial fluid in the joint contains approximately one third of the proteins found in plasma, with albumin as the major protein component [58].

The *in vitro* drug release test of meloxicam liposomes is also performed employing the “diffusion” method following similar procedures for comparison. The difference is that the solution of meloxicam liposomes is added in the upper chamber, and the release medium (PBS) is added in the lower chamber using a limited number of inserts that are well sealed by the semi-permeable membranes at the bottom (Fig. S6). The meloxicam liposomes are diluted about 4-fold with PBS before being added to the upper chamber of the dialysis device, and then immersed in the release medium. Totally, the meloxicam liposomes are diluted about 20-fold, the same dilution ratio as that used in the *in vitro* anti-inflammatory evaluation. It ensures that the meloxicam concentration in the release medium remains much lower than its saturated solubility during drug release test, i.e., under “sink condition”. Generally, the liposomal drug release rate is accelerated under relatively high dilution factors [59]. As expected, MLX-WaterLipo demonstrates a burst release behavior, and 55% of meloxicam has been released at 2 ​h (Fig. S6, pink curve). However, MLX-Ca(AC)_2_Lipo shows a linear sustained release in the first 12 ​h (Fig. S6, blue curve) with the cumulative drug release less than 75%. The same as that observed in the *in vitro* drug release test based on the “reverse diffusion” method, the membrane-controlled rapid drug release property is obtained for MLX-WaterLipo. The sustained drug release behavior of MLX-Ca(AC)_2_Lipo at higher dilution factor further indicates that the drug release of the actively-loaded liposomes is regulated by disassociation of meloxicam-calcium precipitate inside the liposomes. Consequently, it is promising for MLX-Ca(AC)_2_Lipo to achieve sustained drug release following intra-articular injection in the joint, resulting in improved bioavailability and better anti-inflammatory effect.

The storage stability of MLX-Ca(AC)_2_Lipo is evaluated and compared with that of MLX-WaterLipo. It is indicated that the encapsulation efficiency of MLX-WaterLipo drops from 60.6% to 38% after storage for 7 days at 4 ​°C (Fig. S7A). In contrast, MLX-Ca(AC)_2_Lipo exhibits excellent storage stability. The encapsulation efficiency of MLX-Ca(AC)_2_Lipo remains above 95%, and the average particle size after storage for 60 days at 4 ​°C (114 ​± ​1.7 ​nm) is not significantly changed with a narrow distribution (PDI: 0.03), as shown in Figs. S7B and S7C. Generally, intra-articular injections are performed every three months. Based on the sustained drug release performance of the actively-loaded meloxicam liposomes, it is anticipated that they may be used for monthly intra-articular injection. However, it is difficult even for the actively-loaded meloxicam liposomes to achieve sustained drug release for a duration of three months, which is generally practiced for intra-articular injection in clincal. To the best of our knowledge, a different typ of liposome, multivesicular liposomes with the “honeycomb” inner structure and large sizes in dozens of microns (DepoFoam™ liposomes) can achieve monthly or longer drug release after *in situ* injection [60]. Unfortunately, DepoFoam™ liposomes are not suitable for loading poorly water soluble drugs as they are prepared by a water/oil/water double emulsion method [61]. Consequently, it may be possible to achieve sustained drug release for three months by further incorporating the liposomes to hydrophilic matrix made of various polymers. The free meloxicam released from the liposomes need to diffuse through the matrix before entering the synovial fluid, and the hydrophilic matrix can also increase the retention of liposomes in the joints.

**Fig. 3:**
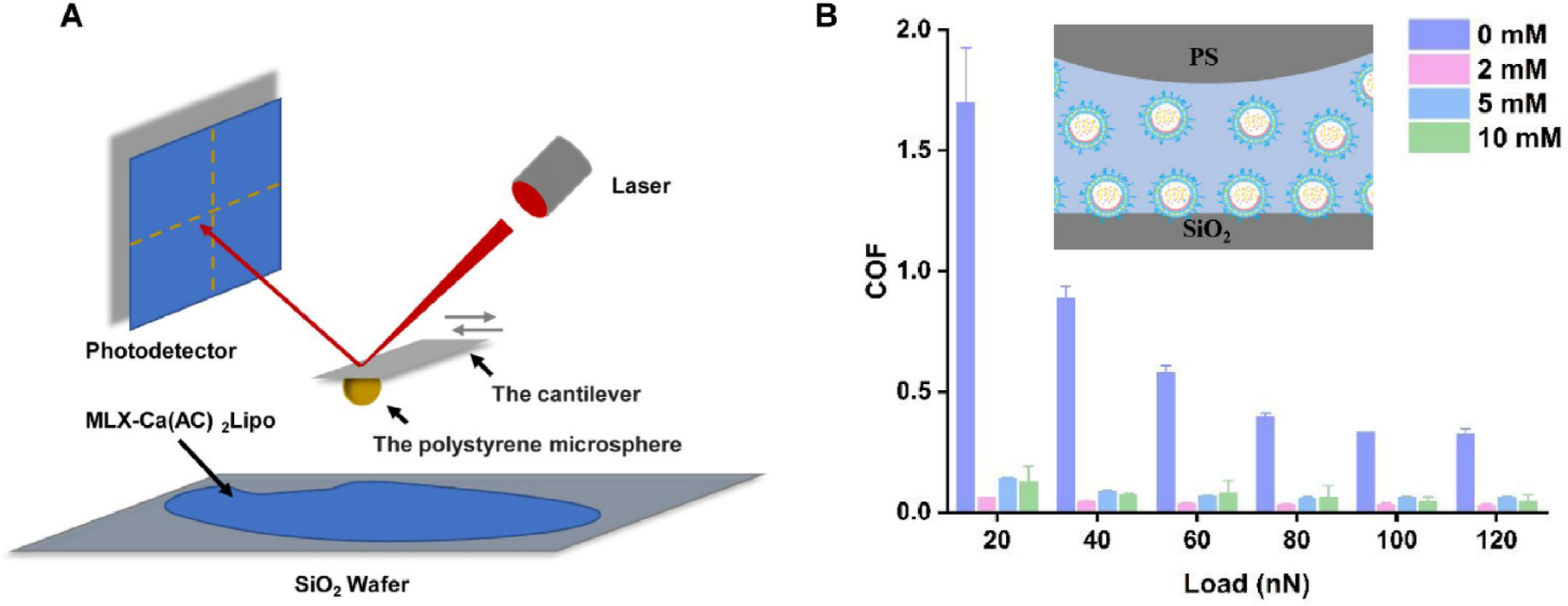
Lubrication performance of meloxicam liposomes. (A) Schematic illustration of the lubrication experimental setup on AFM using meloxicam liposomes as the lubricant. (B) Lubrication performance of MLX-Ca(AC)_2_Lipo under different concentrations and normal loads.

The lubrication dysfunction of articular cartilage is one of the primary reasons for the development of TMJ osteoarthritis, and the increased joint friction can significantly accelerate progression of osteoarthritis [62,63]. It has been reported that phospholipids generate excellent hydration lubrication effect, which is attributed to the hydration layers formed surrounding the positive and negative headgroups [64,65]. In this study, the lubrication performance of MLX-Ca(AC)_2_Lipo is investigated by AFM according to the method proposed in our previous studies [33,34]. The COF value of the liposomes at different concentrations is measured based on the slope of the linear curve of lateral force versus normal load. During lateral force measurement, the probe is pressed against the silicon wafer (a layer of liposomes is adsorbed on the surface) at an applied load, while the silicon wafer slide horizontally underneath the cantilever. To protect the relatively soft liposomes adsorbed on the surface of silicon wafer from being punctured by the sharp tip of probe, a polystyrene microsphere is attached to the probe by UV irradiation. The result of lubrication test indicates that the MLX-Ca(AC)_2_Lipo meloxicam liposomes can effectively reduce the COF value between the silicon wafer and the polystyrene microsphere at the tip of probe (Fig. 3A). At the concentration of 2 ​mM, MLX-Ca(AC)_2_Lipo successfully reduces the COF value to less than 0.05 across the load range of 20∼120 ​nN, as shown in Fig. 3B. In previous studies, the lubrication property of hyaluronic acid (a common viscosupplement intra-articularly injected to the joint to enhance lubrication) has been examined using AFM, and the results show that the COF values of hyaluronic acid are in the range of 0.05–0.168 under different test conditions [34,66,67]. Therefore, the COF value of meloxicam liposomes is similar or even lower than that of hyaluronic acid, indicating that the actively-loaded meloxicam liposomes developed in this study are endowed with excellent lubrication performance. Generally, the enhanced lubrication effect of the meloxicam liposomes following local intra-articular injection is specifically beneficial for protecting articular cartilage from further frictional damage.

### 3.3 Biocompatibility evaluation

**Fig. 4:**
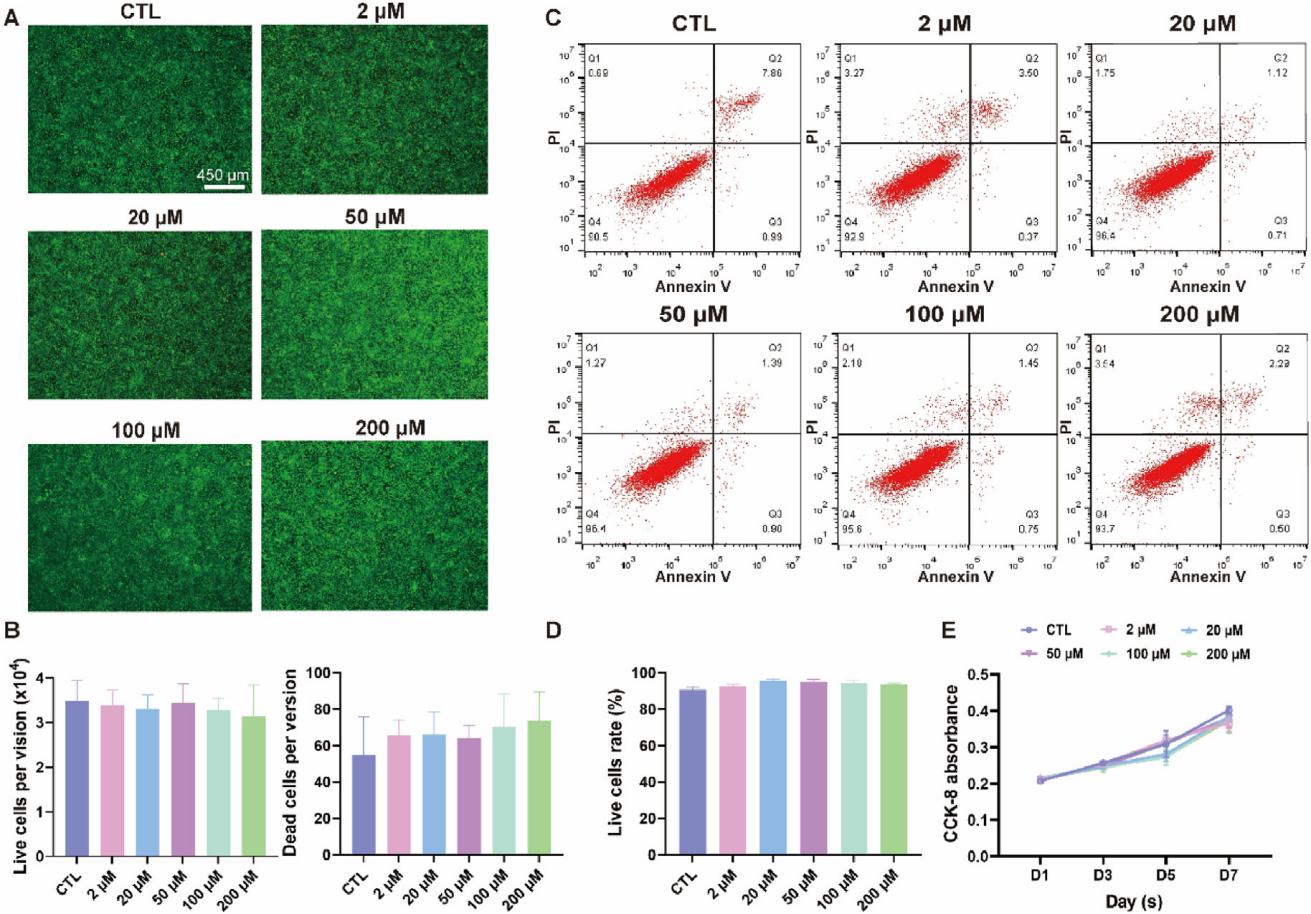
Biocompatibility evaluation of MLX-Ca(AC)_2_Lipo. (A) Live-Dead staining of ATDC5 cells treated with different concentrations of MLX-Ca(AC)_2_Lipo for 24 ​h. (B) Statistical result of ATDC5 cells of the Live-Dead staining assay under single field of view. (C) Flow cytometry analysis of ATDC5 cells treated with different concentrations of MLX-Ca(AC)_2_Lipo for 24 ​h. (D) Statistical result of ATDC5 live cells of the flow cytometry test. Each group shows a high survival rate and a low apoptosis rate (p ​> ​0.05). (E) CCK-8 assay showing proliferation rate of ATDC5 cells treated with different concentrations of MLX-Ca(AC)_2_Lipo for 7 days.

Biocompatibility is one of the significant properties for various biomaterials before clinical application [68]. The biocompatibility evaluation of MLX-Ca(AC)_2_Lipo is characterized at different concentrations and cultured with ATDC5 mouse chondrogenic cell line. As displayed in Fig. 4A and B, the Live-Dead staining assay reveals that the cells are metabolically active, and no obvious cell death is detected for all the liposomal concentrations tested. In addition, the result of flow cytometry shows no significant difference in cell survival rate, apoptosis rate, and death rate for all the groups, indicating that MLX-Ca(AC)_2_Lipo at the concentration from 2∼200 ​μM is biocompatible with the cells (Fig. 4C and D). Similarly, the result of CCK-8 assay exhibits that after 1, 3, 5, and 7 days of co-culturing with MLX-Ca(AC)_2_Lipo at different liposomal concentrations, cell proliferation rate is not significantly affected compared with the control group (CTL, p ​> ​0.05), as shown in Fig. 4E. This indicates that MLX-Ca(AC)_2_Lipo has no obvious *in vitro* cytotoxicity to the cells.

In the present study, Ca^2+^ is used for the preparation of actively-loaded meloxicam liposomes. To ensure that the remaining Ca^2+^ introduced by the intra-articular injection of MLX-Ca(AC)_2_Lip will not produce calcium salt precipitates in the joint of rat after meloxicam is metabolized, calcium acetate (300 ​mM, pH ​= ​6.5) is added to the freshly collected synovial fluid and the transparency of the mixture solution is examined. The apparent calcium concentration in the meloxicam liposomes is 300 ​mM since calcium is only available inside the liposomes. The entrapped volume (i.e., the volume of the space inside the liposomes) of the liposomes (size: 100 ​nm) at the lipid concentration of 20 ​mM is about 2% [69]. That is to say, the liposomal inner phase accounts for 2% of the total volume. Accordingly, the Ca^2+^ introduced after injection of the liposomes into the rat joint cavity is expected to be 6 ​mM, assuming that there is no dilution of the liposomes following intra-articular injection. The Ca^2+^-synovial fluid mixture solution remains transparent, and the formation of calcium salt precipitates is not observed under light microscopy (Fig. S8). The UV absorbance of mixture solution at the wavelength of 600 ​nm is as low as 0.034, which is comparable to that of synovial fluid and purified water (0.035 and 0.021, respectively). The above result indicates that the calcium in the meloxicam liposomes will not lead to the formation of calcium salt precipitates in the rat joint cavity.

### 3.4 In vitro anti-inflammatory evaluation

**Fig. 5:**
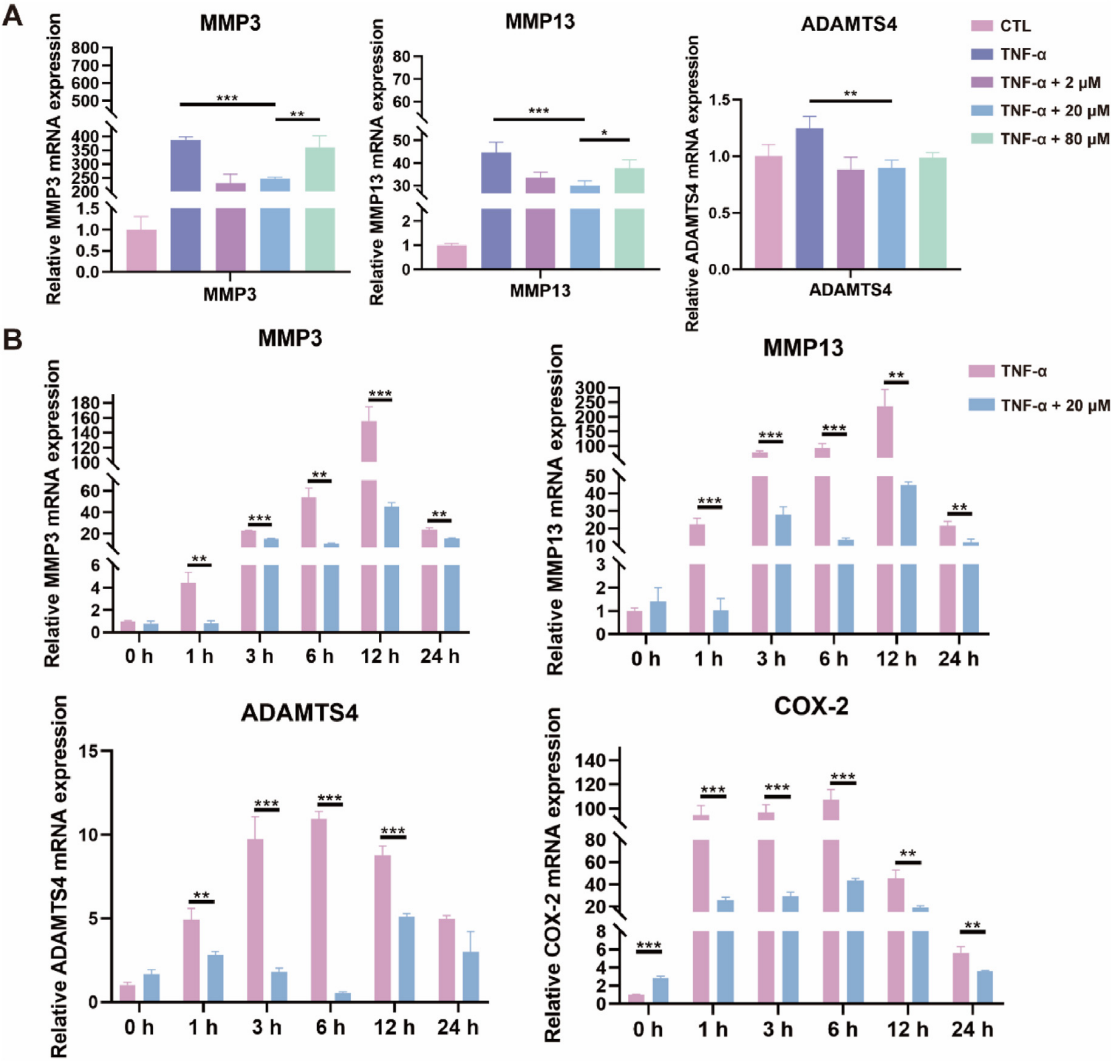
MLX-Ca(AC)_2_Lipo inhibits the mRNA expression levels of cartilage catabolic factors and inflammatory factor *in vitro*. (A) Change of the mRNA expression levels of catabolic factors treated by various concentrations of MLX-Ca(AC)_2_Lipo after TNF-α stimulation. The data are presented as mean ​± ​SD (∗p ​< ​0.05; ∗∗p ​< ​0.01; ∗∗∗p ​< ​0.001). (B) Change of the mRNA expression levels of the biomarkers for cartilage extracellular matrix degeneration (e.g., MMP3, MMP13, and ADAMTS4) and inflammatory factor (i.e., COX-2) with or without MLX-Ca(AC)_2_Lipo treatment at different intervals after TNF-α stimulation. The data are presented as mean ​± ​SD (∗p ​< ​0.05; ∗∗p ​< ​0.01; ∗∗∗p ​< ​0.001).

To determine the optimal concentration of MLX-Ca(AC)_2_Lipo for treatment, three different concentrations (2, 20, and 80 ​μM) are tested for the *in vitro* anti-inflammation effect. It is shown in Fig. 5A that the mRNA expression levels of MMP3, MMP13, and ADAMTS4, which are typical biomarkers for representing the degeneration of cartilage extracellular matrix, are greatly increased after stimulation with TNF-α and decreased after treatment with MLX-Ca(AC)_2_Lipo at different concentrations. Compared with the results of mRNA expression levels for the biomarkers at the liposomal concentration of 2 and 20 ​μM, it seems that the treatment with higher liposomal concentration of 80 ​μM generates a slight increase in cartilage catabolic enzymes. Consequently, the liposomal concentration of 20 ​μM has been selected and employed in the following experiments. Additionally, the mRNA expression levels of the cartilage catabolic enzymes including MMP3, MMP13, and ADAMTS4 increase at different times after TNF-α stimulation of ATDC5 cells for 24 ​h, as shown in Fig. 5B. The adding of MLX-Ca(AC)_2_Lipo at the concentration of 20 ​μM again downregulates the mRNA expression levels. The mRNA expression level of the inflammatory factor (i.e., COX-2) demonstrates a similar trend as mentioned above, and the treatment of MLX-Ca(AC)_2_Lipo at the concentration of 20 ​μM downregulates the mRNA expression level of COX-2 compared with that of TNF-α stimulation group.

**Fig. 6:**
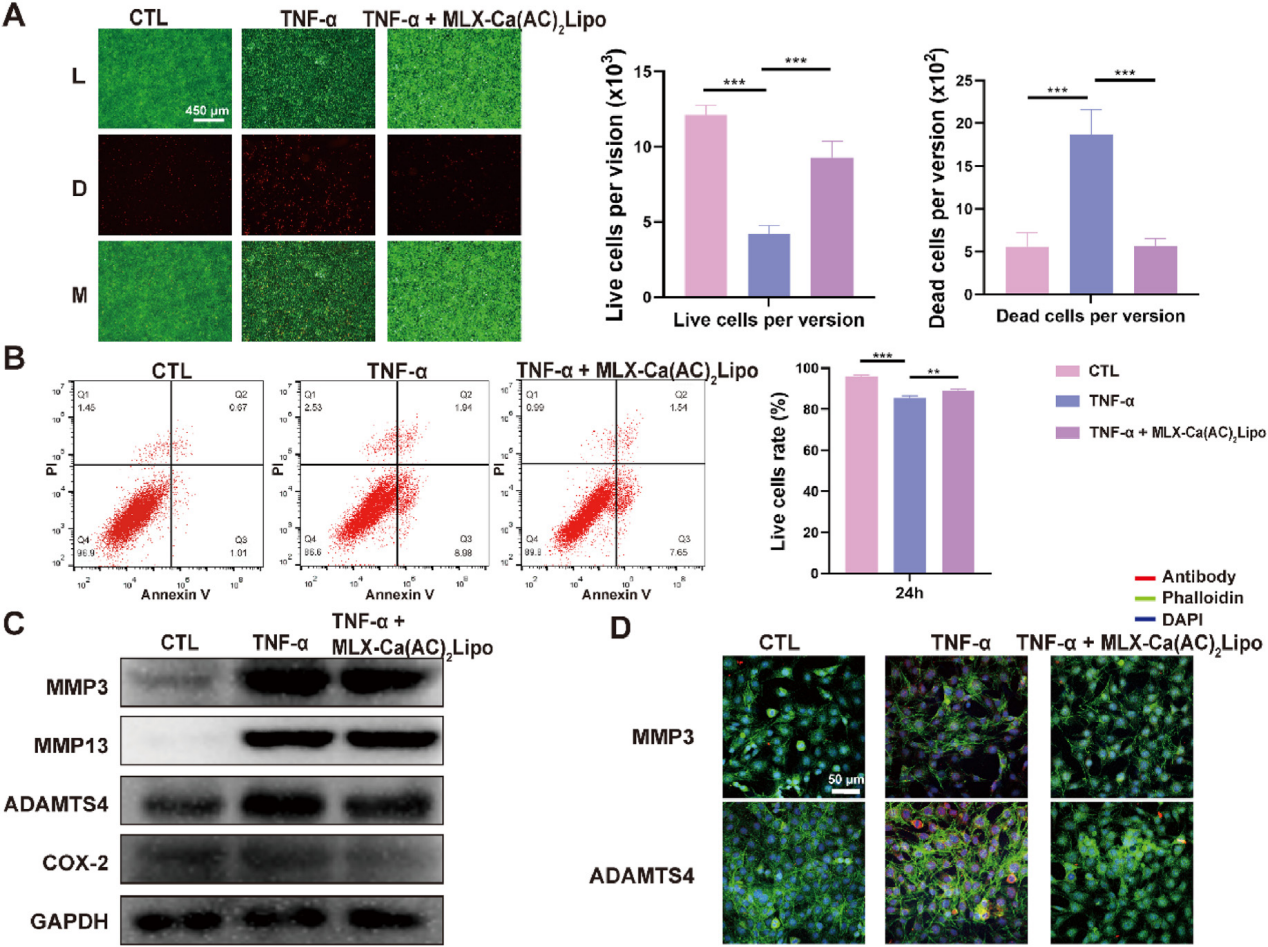
*In vitro* anti-inflammatory characterizations of MLX-Ca(AC)_2_Lipo co-cultured with ATDC5 cells. (A) Live-Dead staining result of control group, TNF-α stimulation group, and TNF-α ​+ ​MLX-Ca(AC)_2_Lipo treatment group and statistical analysis of live and dead cells under single field of view. Green fluorescence indicates live cells, and red fluorescence indicates dead cells. L: live cells; D: dead cells; M: merge (B) Flow cytometry evaluation of control group, TNF-α stimulation group, and TNF-α ​+ ​MLX-Ca(AC)_2_Lipo treatment group. (C) Western blot result demonstrating the treatment of MLX-Ca(AC)_2_Lipo on the protein expression levels of MMP3, MMP13, ADAMTS4, and COX-2 in ATDC5 cells under inflammatory stimulation. (D) Immunofluorescence staining of ATDC5 cells after intervention of MLX-Ca(AC)_2_Lipo under inflammatory stimulation. The data are presented as mean ​± ​SD (∗p ​< ​0.05; ∗∗p ​< ​0.01; ∗∗∗p ​< ​0.001).

The results of Live-Dead staining and flow cytometry demonstrate that the number of dead cells is significantly increased following TNF-α stimulation, and the trend is reversed after treatment of MLX-Ca(AC)_2_Lipo at the concentration of 20 ​μM (Fig. 6 A and **6B**). The result of western blot shows that the protein expression levels of MMP3, ADAMTS4, and COX2 are reduced after treatment of MLX-Ca(AC)_2_Lipo (Fig. 6C). Additionally, immunofluorescence staining demonstrates that the fluorescence intensity of MMP3 and ADAMTS4 is greatly reduced after treatment of MLX-Ca(AC)_2_Lipo (Fig. 6D). Overall, the above results indicate that MLX-Ca(AC)_2_Lipo can effectively inhibit chondrocytes apoptosis and prevent cartilage extracellular matrix degradation under the inflammatory stimulation.

### 3.5 In vivo treatment of TMJ osteoarthritis

**Fig. 7:**
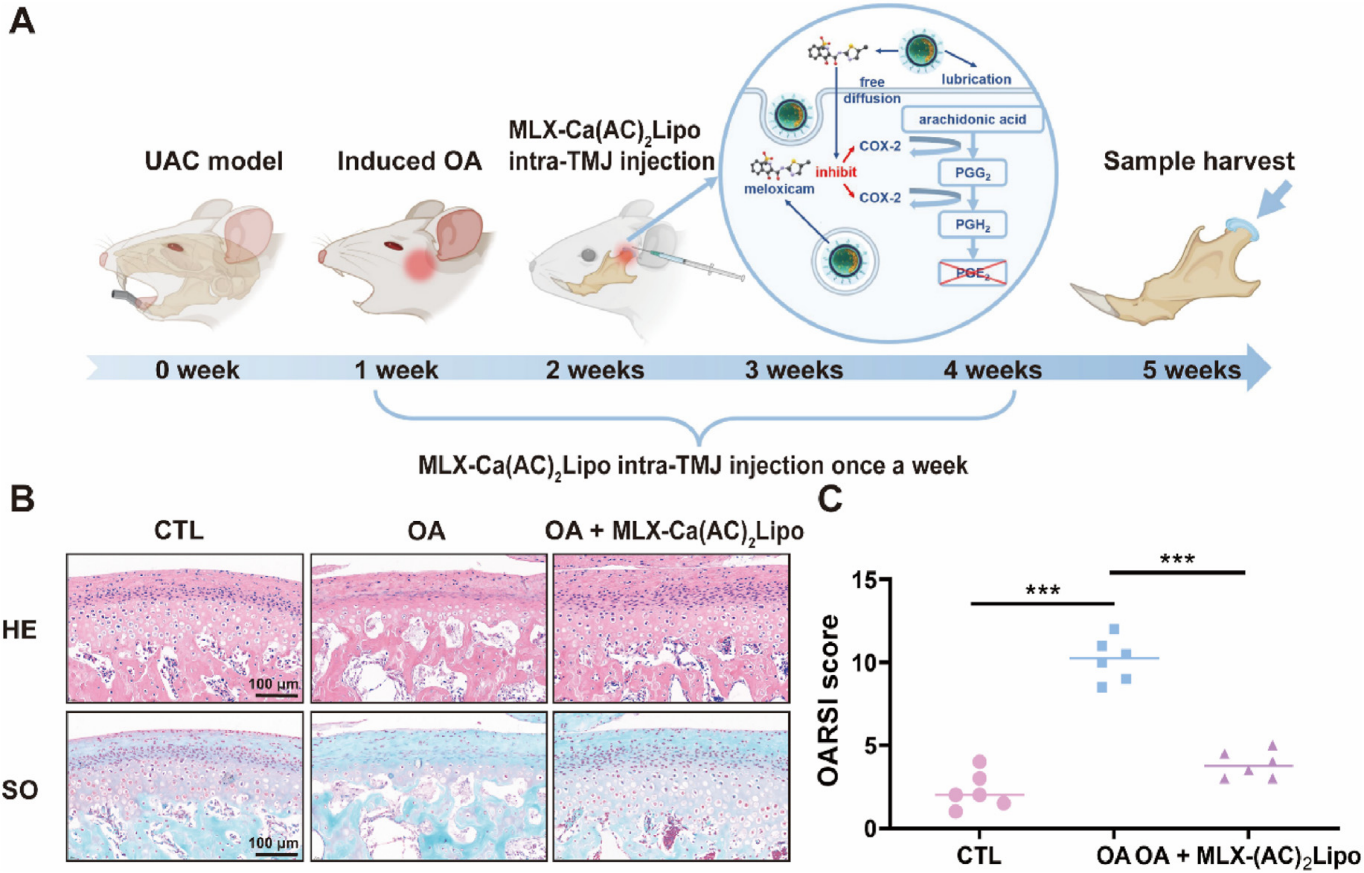
*In vivo* evaluation of MLX-Ca(AC)_2_Lipo for the treatment of TMJ osteoarthritis. (A) Intra-articular injection of MLX-Ca(AC)_2_Lipo in the rats and potential mechanism for achieving anti-inflammation effect. (B) HE and Safranin O staining of rat condylar cartilage sections for different groups. (C) Quantitative analysis of OARSI score based on the staining result. ∗∗∗p ​< ​0.001. CTL: sham operation group; OA (osteoarthritis): unilateral anterior crossbite model group; OA ​+ ​MLX-Ca(AC)_2_Lipo: unilateral anterior crossbite model with MLX-Ca(AC)_2_Lipo local intra-articular injection group.

**Fig. 8:**
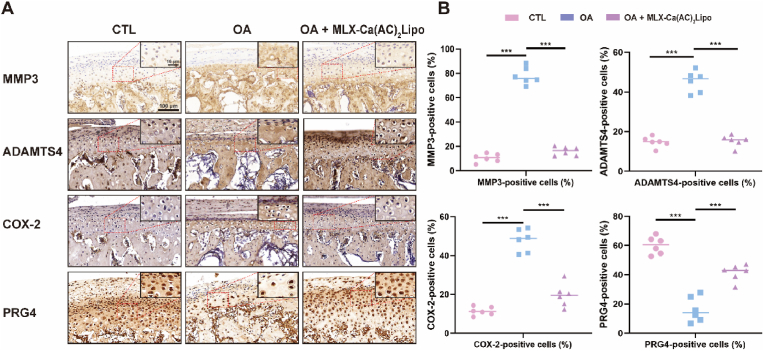
*In vivo* therapeutic outcome of MLX-Ca(AC)_2_Lipo for anti-inflammation effect. (A) Immunohistochemical staining of rat condylar cartilage sections for the analysis of MMP3, ADAMTS4, COX-2, and PRG4 in different groups. (B) Quantitative evaluation of the MMP3, ADAMTS4, COX-2, and PRG4 positive cells for different groups. ∗∗∗p ​< ​0.001. CTL: sham operation group; OA (osteoarthritis): unilateral anterior crossbite model group; OA ​+ ​MLX-Ca(AC)_2_Lipo: unilateral anterior crossbite model with MLX-Ca(AC)_2_Lipo local intra-articular injection group.

The overall procedure for performing the *in vivo* animal test is demonstrated in Fig. 7A, which mainly includes the establishment of UAC rat model, intra-articular injection of MLX-Ca(AC)_2_Lipo, and potential mechanism of the treatment of TMJ osteoarthritis. The rats are fed with soft food to ensure nutrition when the UAC model is established. Before the *in vivo* experiment is performed, methylene blue is used to identify the joint cavity of the rat. The suck-back process is operated to confirm that the needle is in the upper joint space, and then the materials are slowly and gently injected without damage to the surrounding maxillofacial muscles, nerves, and glandular tissues. According to the result of a previous study [70], in which the delivery of drug-loaded liposomes (i.e., cationic liposomes co-loaded with lornoxicam and microRNA-140) demonstrates an improved therapeutic outcome compared to pure liposomes in the treatment of rat knee osteoarthritis, the biological effect of actively-loaded meloxicam liposomes is mainly focused in the *in vivo* test of the present study. It is clearly indicated in Fig. 7B of the HE and Safranin O staining for the rat condylar cartilage sections of various groups that the cartilage and subchondral bone illustrate typical osteoarthritis manifestation after UAC, and the thickness of articular cartilage layer is increased following the treatment of MLX-Ca(AC)_2_Lipo. The OARSI score of the samples in the MLX-Ca(AC)_2_Lipo treatment group is comparable with that of the control group, which is also significantly lower than that of the osteoarthritis group, as demonstrated in Fig. 7C. In addition, the immunohistochemical staining result of the rat condylar cartilage sections for different groups displays that the intra-articular injection of MLX-Ca(AC)_2_Lipo can significantly reduce the positive cell rate of MMP3, ADAMTS4, and COX-2 but meanwhile increase the positive cell rate of proteoglycan 4 (PRG4), as indicated in Fig. 8. As the therapeutic agent that is encapsulated in liposome formulation, meloxicam plays an important role in achieving the anti-inflammation effect by inhibiting the expression level of COX-2, which is a synthase of the inflammatory factor, i.e., prostaglandin E2 (PGE2) [71]. In TMJ osteoarthritis, PGE2 is highly expressed, which induces an upregulation of MMP3 and ADAMTS4 for degradation of the cartilage extracellular matrix [72]. In the present study, MLX-Ca(AC)_2_Lipo injected intra-articularly can inhibit the catabolic activity of chondrocytes based on the sustained release of meloxicam from the liposomes, which is beneficial to the maintenance of chondrocyte homeostasis. MLX-Ca(AC)_2_Lipo also contributes to the increase of the expression level of PRG4, which is an evaluation index for the integrity and lubrication performances of articular cartilage [73]. The enhanced lubrication property of MLX-Ca(AC)_2_Lipo due to the hydration lubrication mechanism of the zwitterionic charges can prevent progressive wear damage of articular cartilage and improve lubrication of the joint.

## 4 Conclusions

In the present study, we designed and developed novel meloxicam liposomes with dual-functions of anti-inflammation and lubrication using the active loading method for the treatment of TMJ osteoarthritis. Specifically, meloxicam-meglumine complex was prepared to increase aqueous solubility of meloxicam, and calcium acetate was used as the transmembrane gradient for achieving active loading. Compared with the liposomes prepared with passive loading method, the actively-loaded MLX-Ca(AC)_2_Lipo resulted in high encapsulation efficiency, good storage stability, and slow drug release properties. The *in vitro* and *in vivo* biological experiments demonstrated that MLX-Ca(AC)_2_Lipo was endowed with good biocompatibility and lubrication, and could effectively inhibit chondrocytes apoptosis and cartilage extracellular matrix degeneration under severe inflammatory conditions. In summary, the actively-loaded MLX-Ca(AC)2Lipo may be a promising candidate for the treatment of TMJ osteoarthritis by intra-articular therapy.

## Author statement

Y.Q. Zhong: Investigation, Methodology, Writing - Original Draft; Y.Y. Zhou: Investigation, Methodology, Writing - Original Draft; R.Y. Ding: Formal analysis, Writing - Review & Editing; L.X. Zou: Formal analysis, Writing - Review & Editing; H.Y. Zhang: Conceptualization, Resources, Funding acquisition, Writing - Review & Editing; X.H. Wei: Conceptualization, Resources, Funding acquisition, Writing - Review & Editing; D.M. He: Conceptualization, Resources, Funding acquisition, Writing - Review & Editing.

## Declaration of competing interest

The authors declare that they have no known competing financial interests or personal relationships that could have appeared to influence the work reported in this paper.

## Data Availability

Data will be made available on request.
