# Supplementary material for: Intra-articular treatment of temporomandibular joint osteoarthritis by injecting actively-loaded meloxicam liposomes with dual-functions of anti-inflammation and lubrication
Source: Mater Today Bio. 2023 Feb 3;19:100573. doi: 10.1016/j.mtbio.2023.100573 (PMC9929446; doi:10.1016/j.mtbio.2023.100573)
Supplement: Multimedia component 1 [file mmc1.docx]

**Supporting Information**

**Intra-articular treatment of temporomandibular joint osteoarthritis by injecting actively-loaded meloxicam liposomes with dual-functions of anti-inflammation and lubrication**

Yingqian Zhong^a,1^, Yuyu Zhou^b,1^, Ruoyi Ding^a^, Luxiang Zou^a^, Hongyu Zhang^c,*^, Xiaohui Wei^b,*^, Dongmei He^a,*^

^a^ Department of Oral Surgery, Ninth People’s Hospital, Shanghai Jiao Tong University School of Medicine; Shanghai Key Laboratory of Stomatology & Shanghai Research Institute of Stomatology; National Clinical Research Center of Stomatology, Shanghai, 200011, China.

^b^ School of Pharmacy, Shanghai Jiao Tong University, Shanghai, 200240, China.

^c^ State Key Laboratory of Tribology, Department of Mechanical Engineering, Tsinghua University, Beijing 100084, China.

^1^ Y.Q. Zhong and Y.Y. Zhou contributed equally to this work.

^*^ Corresponding authors

H.Y. Zhang, Email: zhanghyu@tsinghua.edu.cn

X.H. Wei, Email: xhwei@sjtu.edu.cn

D.M. He, Email: lucyhe119@163.com


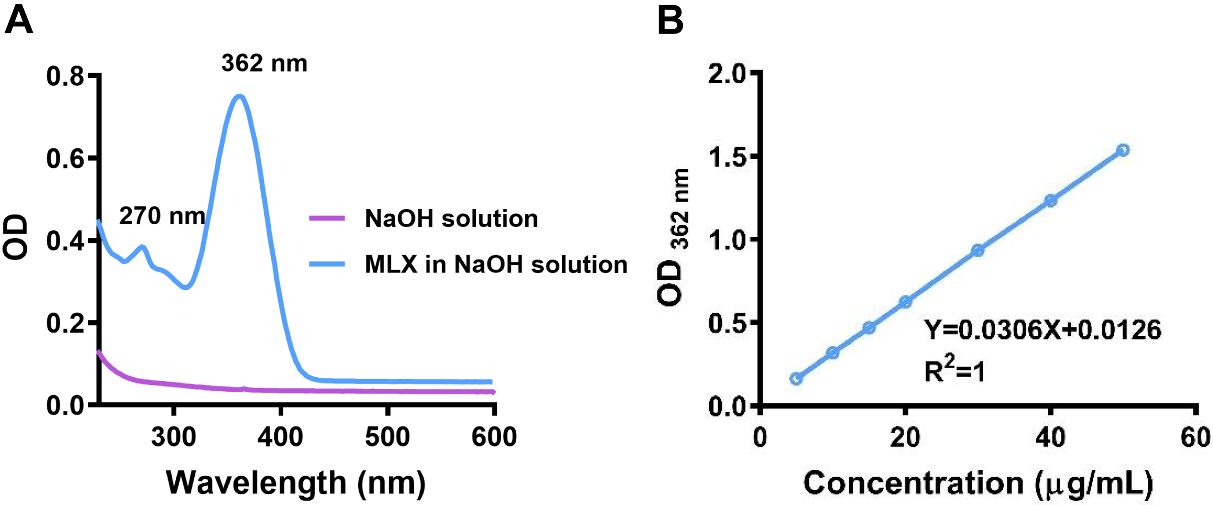


**Fig. S1**. (A) UV spectrum of meloxicam dissolved in NaOH solution. (B) Calibration curve of meloxicam dissolved in NaOH solution by UV absorbance at 362 nm.


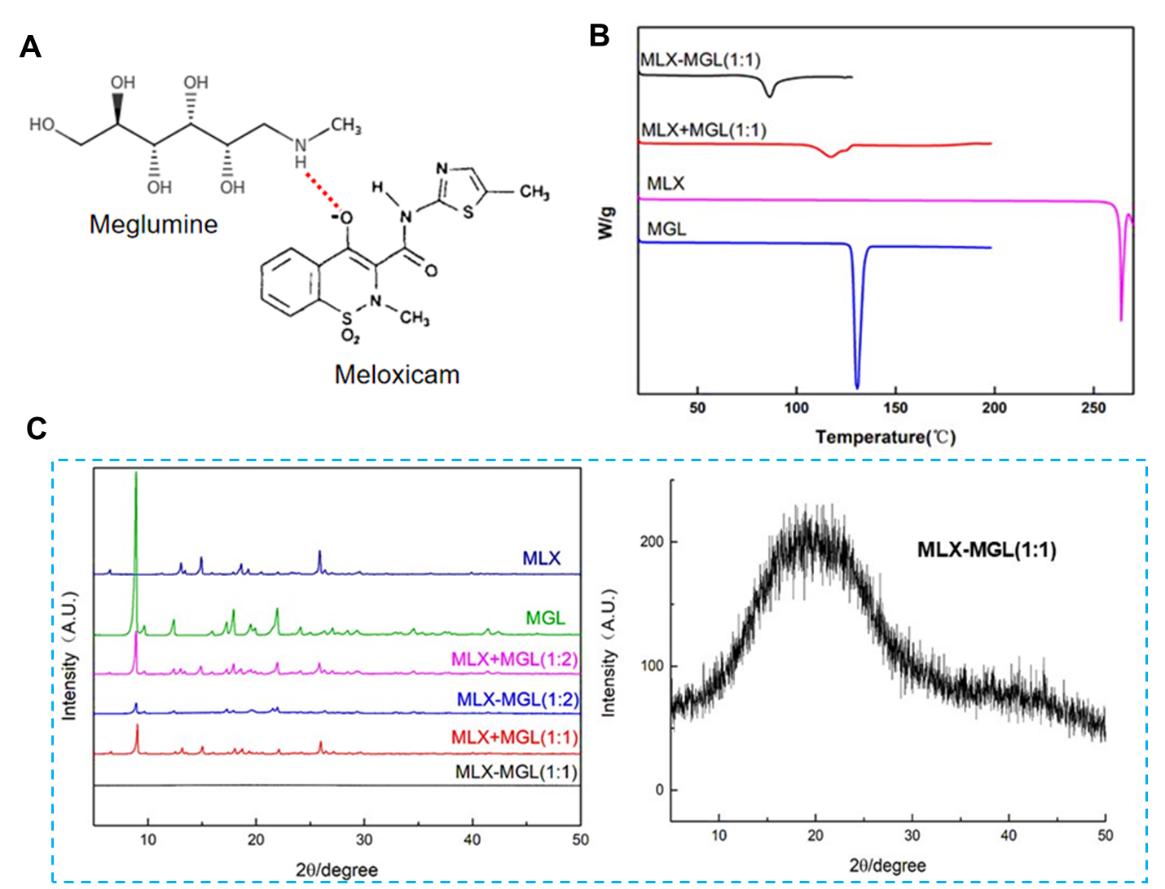


**Fig. S2**. (A) Proposed structure of meloxicam-meglumine complex and (B) differential scanning calorimetry (DSC) & (C) powder X-ray diffraction (XRD) characterizations of the salts at different meloxicam to meglumine molar ratios. In (B) and (C): MLX: meloxicam; MGL: meglumine; MLX-MGL (1:1/1:2): meloxicam-meglumine complex prepared at the molar ratio of 1:1 and 1:2; MLX+MGL (1:1/1:2): physical mixture of meloxicam and meglumine at the molar ratio of 1:1 and 1:2.


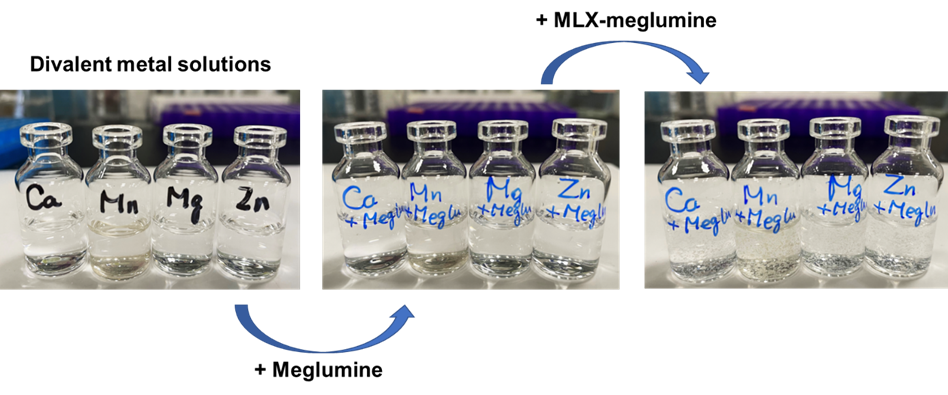


Fig. S3. The mixture of four divalent metal solutions with meglumine and meloxicam-meglumine complex. Left: the four divalent metal solutions used for the preparation of meloxicam liposomes; Middle: the mixture of the four divalent metal solutions with meglumine (100 mg/mL, pH = 7). The concentration of meglumine in the mixtures was 5 mg/mL; Right: the metal meglumine mixture solutions in the middle further mixed with meloxicam-meglumine complex (10 mg/mL, pH = 7). The concentration of MLX in the mixtures was 65 µg/mL.


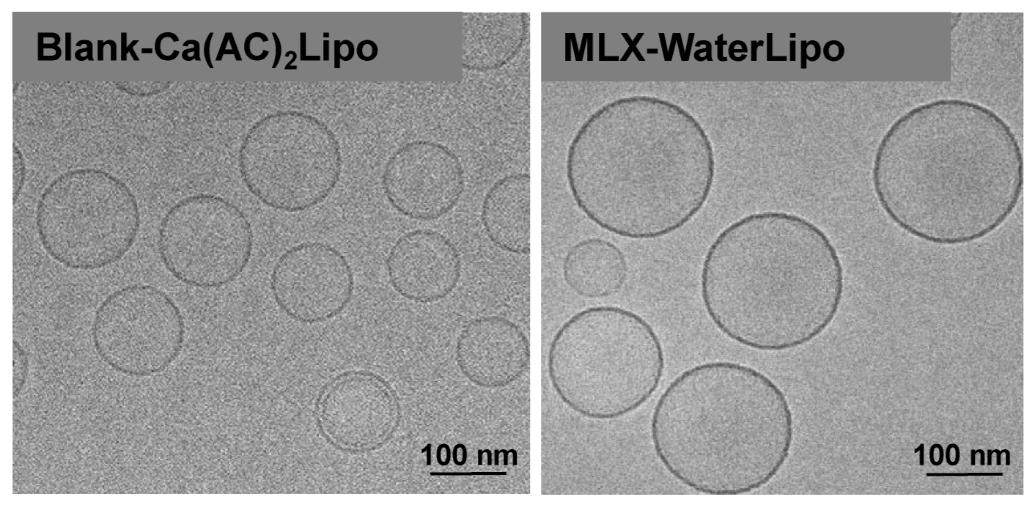


**Fig. S4**. The cryo-TEM images of (A) blank liposomes (B) and MLX-WaterLipo under low magnification.


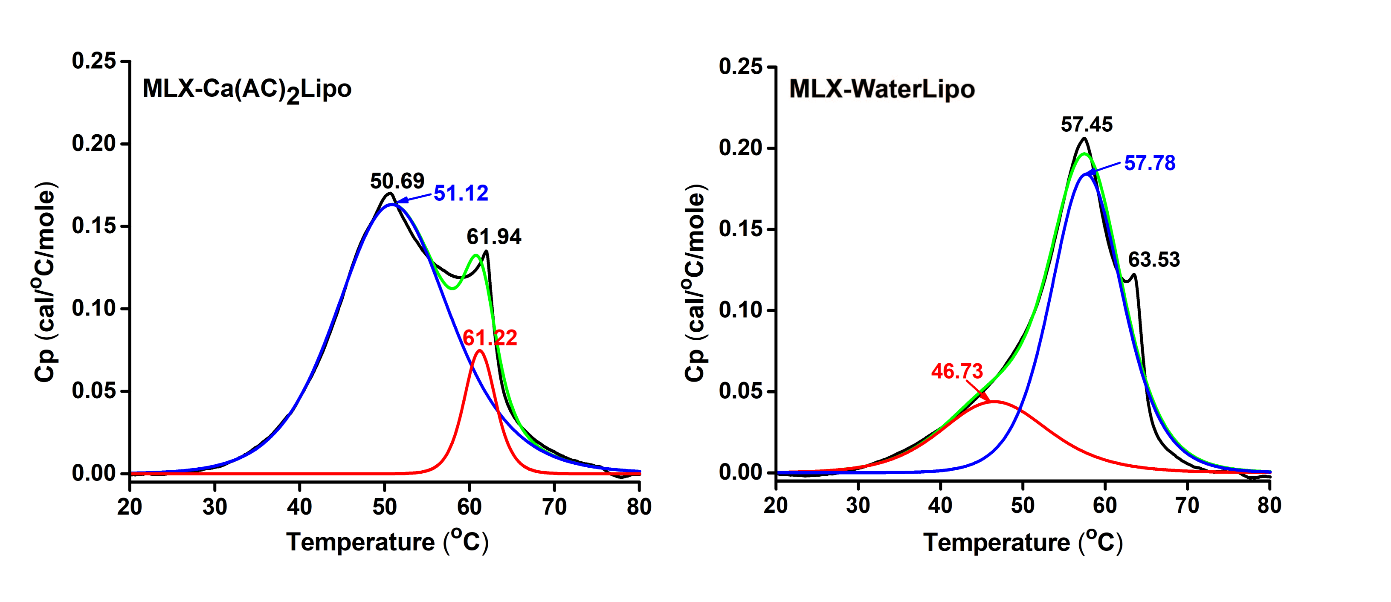


**Fig. S5**. Fitting results of thermograms for the two kinds of meloxicam liposomes (i.e., MLX-Ca(AC)_2_Lipo and MLX-WaterLipo) at minimum chi-square values (black curve: the original thermogram; blue and red curves: the fitted endotherm; green curve: the fitted thermogram). The fitted thermograms overlap well with the original thermograms.


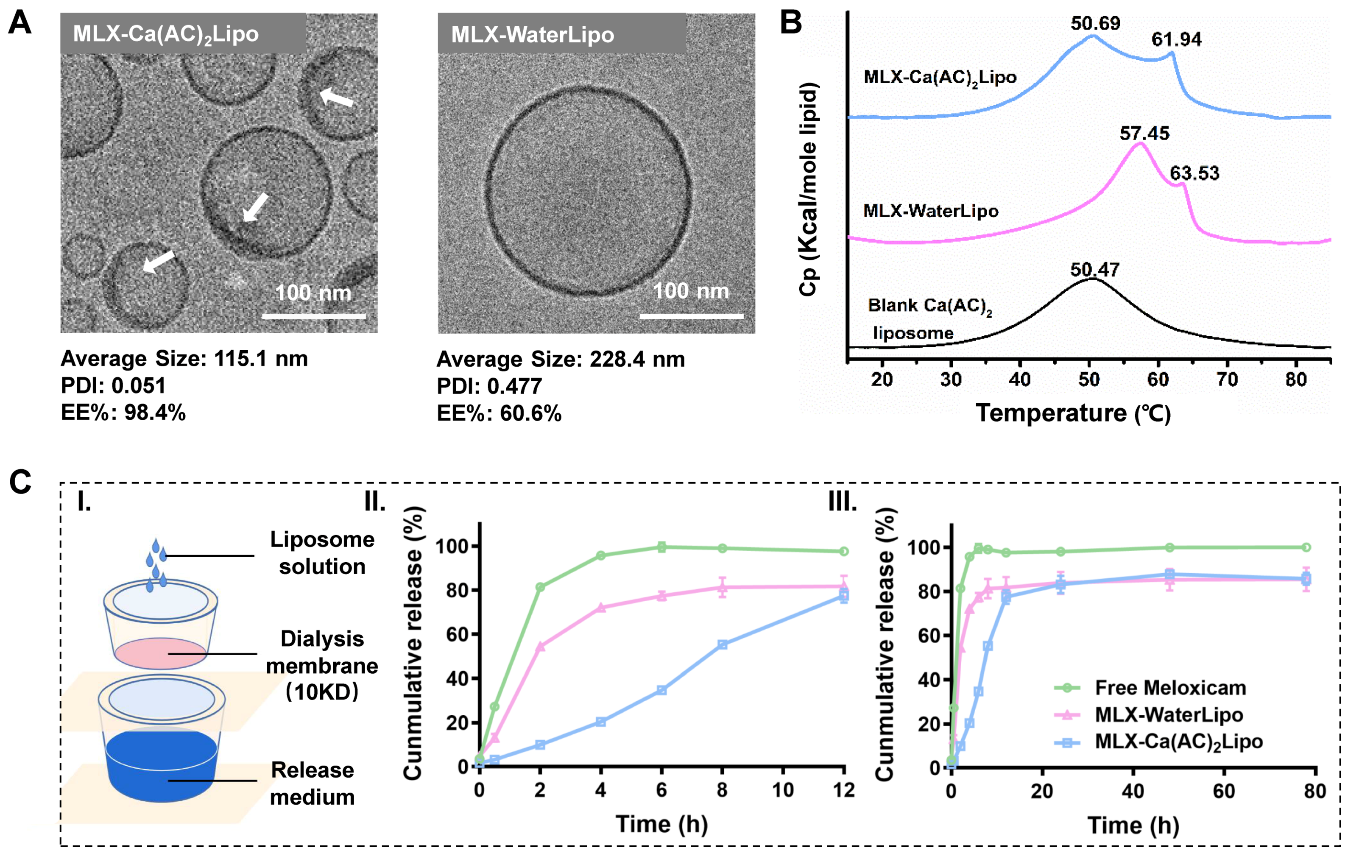


**Fig. S6**. The *in vitro* release behavior of two kinds of meloxicam liposomes over 78 h by the “diffusion” method. (I.) The schematic diagram showing the home-made dialysis device; (II.) The cumulative release curves within the first 12 h and (III.) over 78 h.

The schematic illustration of the home-made dialysis device for performing the drug release test is presented in **Fig. S6-I**. A burst drug release (54.48 ± 1.23%) at 2 h is observed for MLX-WaterLipo (**Fig. S6-II**, pink curve). A linear drug release profile in the first 12 h is observed for MLX-Ca(AC)_2_Lipo (**Fig. S6-II**, blue curve), following the zero-order kinetics (R^2^=0.9875). The drug release of MLX-WaterLipo is typically in an exponential profile that reflects a membrane-controlled passive diffusion process, and it is similar to that of meloxicam solution (**Fig. S6-II**, green curve). The results indicate that drug release of MLX-Ca(AC)_2_Lipo is controlled by disassociation of meloxicam-calcium precipitate inside the liposomes but not just diffusion of meloxicam within the first 12 h. The two kinds of meloxicam liposomes release the drug at a similar rate until up to 85% of meloxicam has been released at 78 h, as shown in **Fig. S6-III**. As the control, 81% of the drug is released from meloxicam solution at the first 2 h and more than 95% releases after 4 h. It indicates that the dialysis membrane does not hinder the diffusion of meloxicam to the release medium.


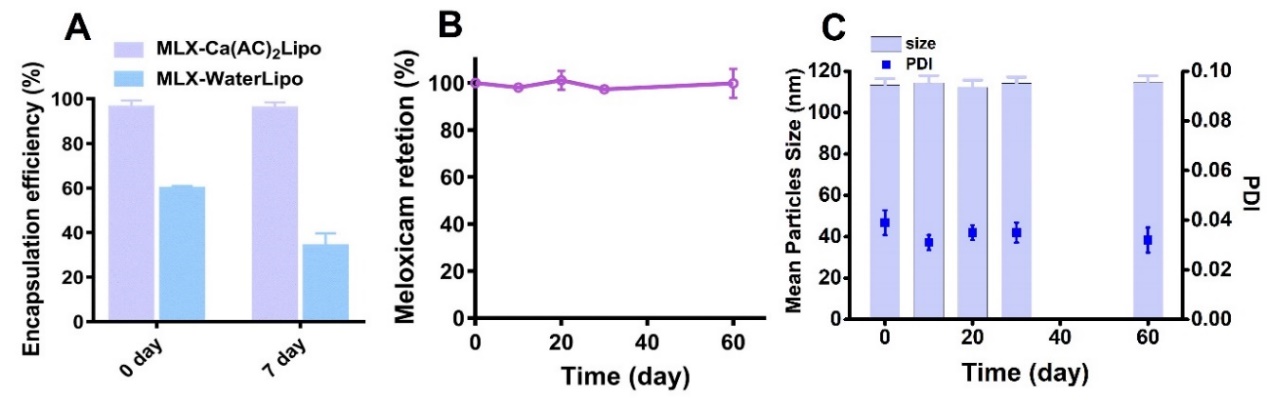


**Fig. S7**. The storage stability of the two meloxicam liposomes (i.e., MLX-Ca(AC)_2_Lipo and MLX-WaterLipo) at 4ºC in 7 days (A) and 60 days (B and C, MLX-Ca(AC)_2_Lipo). (B): Encapsualtion efficienty during storage; (C): Mean particle size and polydispersity index (PDI).


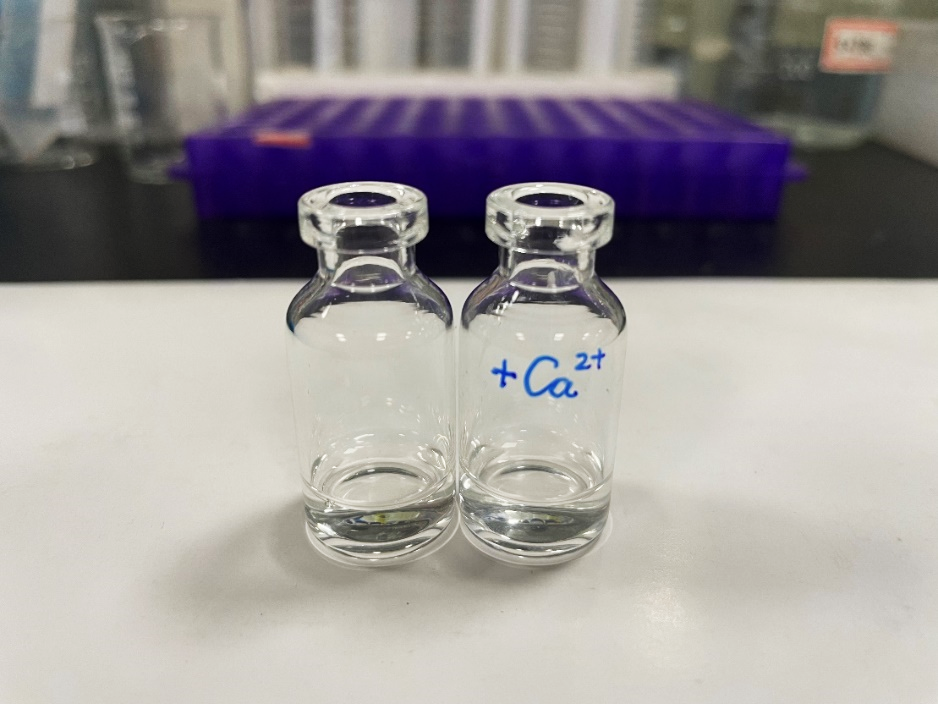


**Fig. S8**. Fresh synovial fluid from temporomandibular disorder (TMD) patients before (left) and after (after) being mixed with calcium acetate.

**Table S1**. Thermodynamic parameters of the blank calcium acetate liposome and two kinds of meloxicam liposomes. The endotherms with T_m_ of around 50ºC are attributed to the lipid-related phase transition and normalized with the lipid concentration of the samples.

| Sample name | Drug conc. (mg/mL) | T_m_1 (ºC) | ΔT_1/2_ (ºC) | ΔH  (kcal/mol) | T_m_2 (ºC) | ΔT_1/2_ (ºC) | ΔH  (kcal/mol) |
| --- | --- | --- | --- | --- | --- | --- | --- |
| Blank liposome | / | 50.47 | 15.67 | 2.35 | / | / | / |
| MLX-Ca(AC)_2_Lipo | 0.9 | 51.12 | 15.76 | 2.45 | 61.22**^a^** | 4.42**^a^** | 0.37**^a^** |
| MLX-WaterLipo | 0.38 | 46.73 | 15.45 | 0.78 | 57.78**^b^** | 10.34**^b^** | 2.26**^b^** |

a and b: the data are normalized and calculated with meloxicam and lipid concentration, respectively.

The three thermodynamic parameters including T_m_, ΔT_1/2_, and ΔH in **Table S1** are generated by fitting the original thermograms (**Fig. S5**) with non-two-state model using the software of the DSC instrument. T_m_ refers to the phase transition temperature. For drug-free liposome, T_m_ is the phase transition temperature of phospholipid bilayer from the gel state (solid ordered phase) to the liquid crystalline state (liquid disordered phase) [1]. The value of T_m_ can be shifted from the “peak” value picked up directly from the thermogram according to the model used in the fitting process. ΔT_1/2_ is the width of the transition at half height of the endotherm or exotherm. It represents the cooperativity of the phase transition. Generally, highly ordered structures such as crystals have a small ΔT_1/2_ value in the phase transition. ΔH is the enthalpy of the phase transition, and it is calculated based on the peak area of the endotherm or exotherm. The phase transition of ordered structures is generally accompanied with a large energy input or output, i.e., a large ΔH can be observed for organized structures. The ΔH of lipid membrane phase transition is owing to the required energy for the *trans–gauche* conformational change of the hydrocarbon chains of phospholipids (chain melting) in the liposome membrane. The incorporation of cholesterol into the gel phase increases the fluidity of liposome membrane, resulting in the decrease in ΔH of the main transition. Detailed information on liposomal DSC characterization is available in previous literature [2].

In this study, we used DSC to investigate the loading mechanism of calcium acetate-meloxicam liposomes and potential physical state of the complex. As expected, blank (drug-free) calcium acetate liposomes demonstrated a “weak and broad” endotherm in the heating scan from 15 to 85ºC, typically for cholesterol-rich liposomes [3], and two kinds of endotherms were observed for the meloxicam liposomes. We applied the non-two-state model to fit the two thermograms, and evaluated the different thermodynamic behaviors of the meloxicam liposomes. As shown in **Fig. S5**, the thermogram of the actively-loaded meloxicam liposomes was composed of two phase transitions. One was the lipid membrane changed from the solid disordered phase to liquid ordered phase at around 50ºC, the same as that of the blank liposomes. The other endotherm with a small ΔT_1/2_ may be attributed to the melting of calcium acetate-meloxicam at 61ºC. Therefore, we considered that the actively-loaded liposomes could maintain the same membrane structure as that of the blank liposomes (which is very important for the membrane to keep its “barrier” effect during the process of sustained drug release). By contrast, the fitting thermogram of the passively-loaded liposomes showed a very “weak and broad” phase transition at around 47ºC, indicating that the adding of meloxicam to the liposome membrane could loosen the membrane structure and thus the liposomes became leaky. The small “shoulder” peak at about 63ºC in the thermogram was discarded in the fitting process because of its low enthalpy. Instead, an endotherm with T_m_ of around 58ºC was observed.

**References**

[1] H. Kitayama, Y. Takechi, N. Tamai, H. Matsuki, C. Yomota, H. Saito, Thermotropic phase behavior of hydrogenated soybean phosphatidylcholine–cholesterol binary liposome membrane, Chem. Pharm. Bull. 62 (2014) 58-63.

[2] C. Demetzos, Differential scanning calorimetry (DSC): a tool to study the thermal behavior of lipid bilayers and liposomal stability, J. Liposome Res. 18 (2008) 159-173.

[3] X. Wei, R. Cohen, Y. Barenholz, Insights into composition/structure/function relationships of Doxil^®^ gained from "high-sensitivity" differential scanning calorimetry, Eur. J. Pharm. Biopharm. 104 (2016) 260-270.
